# Effectiveness of an App-Based Short Intervention to Improve Sleep: Randomized Controlled Trial

**DOI:** 10.2196/39052

**Published:** 2023-03-21

**Authors:** Bianka Vollert, Luise Müller, Corinna Jacobi, Mickey Trockel, Ina Beintner

**Affiliations:** 1 Department of Clinical Psychology and Psychotherapy Faculty of Psychology Technische Universität Dresden Dresden Germany; 2 Department of Psychiatry and Behavioral Sciences Stanford University School of Medicine Stanford, CA United States

**Keywords:** sleep, insomnia, cognitive behavioral treatment for insomnia, eHealth, mobile app

## Abstract

**Background:**

A growing body of evidence for digital interventions to improve sleep shows promising effects. The interventions investigated so far have been primarily web-based; however, app-based interventions may reach a wider audience and be more suitable for daily use.

**Objective:**

This study aims to evaluate the intervention effects, adherence, and acceptance of an unguided app-based intervention for individuals who wish to improve their sleep.

**Methods:**

In a randomized controlled trial, we evaluated the effects of an app-based short intervention (Refresh) to improve sleep compared with a waitlist condition. Refresh is an 8-week unguided intervention covering the principles of cognitive behavioral therapy for insomnia (CBT-I) and including a sleep diary. The primary outcome was sleep quality (insomnia symptoms) as self-assessed by the Regensburg Insomnia Scale (RIS). The secondary outcomes were depression (9-item Patient Health Questionnaire [PHQ-9] score) and perceived insomnia-related impairment.

**Results:**

We included 371 participants, of which 245 reported poor sleep at baseline. About 1 in 3 participants who were allocated to the intervention group never accessed the intervention. Active participants completed on average 4 out of 8 chapters. Retention rates were 67.4% (n=250) at postassessment and 57.7% (n=214) at the 6-month follow-up. At postintervention, insomnia symptoms in the intervention group had improved more than those in the waitlist group, with a small effect (d=0.26) in the whole sample and a medium effect (d=0.45) in the subgroup with poor sleep. Effects in the intervention group were maintained at follow-up. Perceived insomnia-related impairment also improved from pre- to postassessment. No significant intervention effect on depression was detected. Working alliance and acceptance were moderate to good.

**Conclusions:**

An app-based, unguided intervention is a feasible and effective option to scale-up CBT-I-based treatment, but intervention uptake and adherence need to be carefully addressed.

**Trial Registration:**

ISRCTN Registry ISRCTN53553517; https://www.isrctn.com/ISRCTN53553517

## Introduction

Sleep is of vital importance for our everyday functioning, health, and well-being [1]. However, difficulties falling or staying asleep are common among the general population worldwide [2-4]. Approximately 1 in 3-4 adults experience symptoms of insomnia, while up to 10%-15% cross the threshold to full-syndrome insomnia [5-7]. Poor sleep is associated with disabling daytime consequences including fatigue, reduced performance, irritability, mood disturbances, and impaired health-related quality of life [8-11].

Individuals with insomnia often report symptoms that persist over several months, indicating a chronic course of insomnia. In a longitudinal study, more than 70% of the participants experienced insomnia for at least one year and 1 in 2 reported that symptoms lasted even longer [12]. Insomnia symptoms can be perpetuated by dysfunctional cognitions and maladaptive sleep-related behaviors and habits that contribute to a psycho-physiological hyperarousal that is incompatible with sleep [13,14].

Insomnia has been linked to various health risks including cardiovascular disease, high blood pressure, and diabetes [15-17]. Poor sleep also has effects on the brain and cognition; impairments in performance, concentration, working memory, and response time are associated with chronic sleep disorders [18-20]. Insomnia also increases the risk for other mental disorders, especially depression [21-23].

Because of substantial direct (eg, medication and increased health care utilization) and indirect costs (eg, absenteeism, reduced work productivity, and accidents), insomnia is associated with a high socioeconomic burden [24-26].

Given the high prevalence, associated health risks, and the global burden, it is crucial that insomnia is recognized early and treated adequately [14]. Cognitive behavioral therapy for insomnia (CBT-I) is consistently deemed the first choice of treatment for insomnia [27,28]. It is considered more suitable than a (pure) pharmacological treatment, especially given the limited evidence for long-term effects of the latter [29]. CBT-I consists of several components including psychoeducational, cognitive, and behavioral elements [30].

CBT-I has shown to be effective in reducing temporary as well as persisting insomnia symptoms in several studies and meta-analyses (eg, [31-36]). In addition, studies suggest that CBT-I improves (comorbid) depression and may even prevent the onset of depression [37-40].

However, reach and access to face-to-face, therapist-delivered CBT-I is limited [41-43], due to both structural and attitudinal barriers. Structural barriers include limited treatment availability, inadequate screening and referrals, and long waiting times [41]. Within-person attitudinal barriers include reservations about help-seeking in general or nonpharmacological treatment options in particular [44,45].

Low-threshold and scalable CBT-based self-help interventions may be a means to overcome these barriers and facilitate access to adequate treatment [46], especially if they are provided online [47]. Internet-based interventions have become increasingly popular in the prevention and treatment of mental disorders (eg, [48]), and offer easy accessibility, flexibility, and anonymity. They allow users to integrate an intervention into daily life and in many cases also to automatically monitor progress [49].

There is a growing body of evidence for internet-based CBI-I interventions [50,51]. Meta-analyses revealed large improvements in insomnia severity (g=1.09) and other sleep outcomes (eg, sleep efficiency and total sleep time) that are comparable to those found in face-to-to interventions [52,53]. Besides, quality of life, executive functions, and work-related health and productivity have shown to be improved through internet-based CBT-I [54-57]. Findings indicate that internet-based CBT-I also prevents or reduces symptoms of depression [58-61].

Fully automated internet-based self-help interventions without human guidance may have the greatest potential when it comes to scalability and cost-effectiveness. While some studies suggest that guided interventions are more effective than unguided ones [53,62], the latter may still yield large and sustainable effects compared with a waitlist or active control condition [63-68].

So far, most research on online interventions has focused on web-based interventions [65]. In the past decade, mobile internet use has been growing and interventions that can be accessed on a smartphone promise a higher potential to reach users than those that require a large screen [69]. By contrast, mobile use may be associated with different user requirements and expectations, including the length and duration of treatment modules or the presentation of information, which may affect adherence and treatment effects.

While there are already a large number of consumer-targeting apps addressing and tracking sleep (behavior) available in mobile app stores, most of them lack evidence [70]. We only identified 2 randomized controlled trials evaluating app-based interventions for individuals with insomnia. One compared a fully automated Dutch intervention focusing on sleep restriction and relaxation with a waitlist condition [65]. The other compared a Persian self-help intervention based on a combination of Theory of Planned Behavior, Health Action Process Approach, and CBT-I with an active control condition (patient education) [71]. Although both interventions did not require any human input, the app by Horsch et al [65] included a conversation tool (chatbot) between the app and the participants as well as a number of persuasive strategies. Both randomized controlled trials applied strict inclusion criteria for study participation (ie, insomnia symptoms in accordance with the criteria for a DSM-5 [Diagnostic and Statistical Manual of Mental Disorders, 5th edition] diagnosis of insomnia) and found that the app-based intervention was superior to the control condition in the improvement of insomnia symptoms.

Given the relative lack of evidence for mobile CBT-I interventions, the aim of this study is to evaluate an unguided app-based training for individuals who wish to improve their sleep. The primary aim is to evaluate the effect of the intervention on sleep quality (insomnia symptoms). Secondary, exploratory aims include the intervention effects on insomnia-related impairment and depression symptoms as well as participant adherence to the intervention, working alliance, and intervention acceptance.

## Methods

### Study Design

Following an uncontrolled feasibility pilot study with 189 participants (S. Buntzel, unpublished data, 2018), we conducted a randomized controlled trial comparing an intervention group with access to a mobile CBT-I intervention with a waiting list control group (Multimedia Appendix 1). Outcomes were assessed through online questionnaires at baseline (pretreatment), postintervention (8 weeks after randomization), and 6-month follow-up. The primary outcome was the change in sleep quality (insomnia symptoms and Regensburg Insomnia Scale [RIS] total score) at postassessment compared with the baseline assessment.

Participants were randomized in a 1:1 ratio. The randomization was block stratified by gender (female vs male); severity of insomnia symptoms, poor (RIS total score ≥13 [72]) versus good sleep (RIS total score <13); and the consumption of sleep-inducing drugs (yes vs no). The random allocation sequence was implemented on the data collection platform.

Participants received information about their group assignment by email. Participants in the intervention group had immediate access to the intervention. By contrast, participants in the waiting list group received access to Refresh after the 6-month follow-up had been completed if they were still interested in using the intervention. Participants in both groups were invited to the post- and follow-up assessments via emails. They received up to 5 reminders at intervals of 3 days.

### Ethics Approval

The study was approved by the Ethics Committee (Institutional Review Board) at Technische Universität Dresden (reference number EK 111032919) and registered prospectively in the ISRCTN database (registration number ISRCTN53553517).

### Inclusion and Exclusion Criteria

We included participants over 18 years of age who were fluent in German and had access to the internet during the intervention period. Exclusion criteria were (1) current treatment for depression, (2) a history of psychotic or bipolar disorders, and (3) a suicidal ideation according to the answer in the last item of the 9-item Patient Health Questionnaire (PHQ-9). Poor sleep was not necessary to participate in the study. All adults showing interest in improving their sleep were welcome if none of the exclusion criteria were present.

### Recruitment/Procedure

Participants were recruited through flyers, postcards, and posters in several sleep laboratories in Germany as well as in practices of general practitioners, pharmacies, and medical supply stores in Dresden. In addition, recruitment activities took place at Technische Universität Dresden and included a press release, student email newsletters as well as postcards and posters distributed on the campus. A newspaper article was published in a regional newspaper. Social media channels (eg, Facebook groups dealing with sleep issues) were used to address further potential participants. The recruitment material included a link and a QR code leading to the study website. Interested participants received written information about the study and informed consent was obtained online. Study data were collected and managed using REDCap (Research Electronic Data Capture; Vanderbilt University) electronic data capture tools hosted at the Centre for Clinical Studies at Technische Universität Dresden. REDCap [73] is a secure, web-based application designed to support data capture for research studies, which provides (1) an intuitive interface for validated data entry; (2) audit trails for tracking data manipulation and export procedures; (3) automated export procedures for seamless data downloads to common statistical packages; and (4) procedures for importing data from external sources.

### Intervention

Refresh is an unguided, fully automated app-based intervention adapted and translated from an e-mail-delivered CBT for sleep health program for college students, which was originally developed and evaluated in the United States [74], and subsequently translated and evaluated in a Japanese college student population [75]. The intervention was adapted for the general adult population and designed as an app-based self-help intervention. Refresh was implemented using a commercially available eHealth platform [76]. Some screenshots of Refresh are provided in Multimedia Appendix 2.

The intervention consists of 8 consecutive chapters that can be completed in about 10 minutes each and cover all CBT-I components recommended by the German Sleep Society (DGSM): psychoeducation, sleep hygiene, stimulus control, sleep restriction, cognitive restructuring, and relaxation [30]. Table 1 summarizes the content of the individual chapters.

**Table 1:** Content of the Refresh intervention.

Chapter	Content
1	Introduction Psychoeducation: (1) rapid eye movement/non–rapid eye movement sleep and stages of sleep; (2) consequences of lack of sleep Introduction to the “30-second sleep diary”
2	Psychoeducation: (1) sleep-wake cycle and the circadian clock; (2) the 2-process model of sleep [77] Establishing a regular sleep schedule
3	Introduction to sleep restriction (only for people with poor sleep) Relaxation: (1) simple breathing relaxation exercise
4	Mindfulness: (1) mindful breathing; (2) body scan
5	Stimulus control Finding compromise: (1) neighbors, spouses, babies Sleep restriction Review of the sleep schedule
6	Rumination and worries: (1) strategies to reduce nighttime rumination; (2) take a mindfulness-based approach to worry
7	Cognitive reappraisal Sleep restriction: (1) review of the sleep schedule
8	Sleep restriction: (1) review of the sleep schedule Wrap up

The chapters are multimedia based with short text passages, audio and video content, vignettes, and questions (multiple choice or free text) to foster an active engagement with the intervention content as well as to tailor subsequent content based on the participants’ preference (eg, whether they preferred information to be presented as text or video). The number of pages per chapter and the amount of plain text per page were kept to a minimum as factors such as extensive text content and text content complexity are likely to increase the risk of nonadherence [78]. The intervention is supplemented by a “30-second sleep diary” to be filled in every morning to monitor insomnia symptoms. For a duration of 8 weeks, an automated reminder to fill in the sleep diary was sent every morning at 7 AM. There were no reminders for progressing through the intervention. At the beginning, participants received a short automated feedback about their sleep based on the baseline RIS score. Participants reporting poor sleep were especially encouraged to use parts of the intervention that were marked as for “people with poor sleep” (eg, sleep restriction).

The intervention is self-paced, but the recommended duration was 8 weeks with 1 chapter per week.

### Measures/Outcomes

#### Regensburg Insomnia Scale

Sleep quality (insomnia symptoms) was assessed using the German RIS [72]. The scale covers psychophysiological (cognitive, emotional, and behavioral) aspects of insomnia during the previous 4 weeks. The instrument consists of 10 items to be answered on a 5-point self-rating scale ranging from 0 to 4. A total score of ≥13 indicates poor sleep [72]. The RIS has shown to be a well-accepted, valid instrument that has discriminative power and is sensitive to detect improvements in insomnia parameters after CBT-I [72].

An additional item asked the participants about their perceived insomnia-related impairment in the past 7 days on a scale ranging from 0 to 100.

#### The 9-Item Patient Health Questionnaire

Depressive symptoms and depression severity were measured using the German version of the PHQ-9 [79]. This widely used brief self-report instrument consists of 9 items covering the DSM-5 diagnostic criteria of depression that are scored on a 4-point Likert scale from 0 (not at all) to 3 (nearly every day). The PHQ-9 has been shown to have good psychometric properties [80] and to be sensitive to changes in depression symptoms over time [81].

#### Working Alliance Inventory-Short Revised

Acceptance of the intervention was assessed using the subscales “task” and “goal” of the Working Alliance Inventory-Short Revised (WAI-SR) [82,83], adapted for online interventions. The inventory is based on Bordin’s Alliance Theory [84]. The “task” subscale (4 items) measures the agreement on the tasks of the intervention, whereas the “goal” subscale (4 items) measures the agreement on the goals of the intervention. Because of the unguided nature of the study, the “bond” subscale (measuring the quality of an affective bond between patient [participant] and therapist [coach]) was removed. Items are answered on a scale from 1 (seldom) to 5 (always), but participants could also choose the additional answer category “I don’t know.” In addition to the WAI-SR, each chapter of the intervention could be rated on a 5-point scale.

#### Adherence Markers

Adherence to the intervention was defined as the number of chapters completed and the proportional progress through the intervention (in percentage). In addition, the number of entries in the sleep diary was used to describe the usage of the intervention.

### Sample Size Calculation

Based on results from the pilot study (S. Buntzel, unpublished data, 2018), we assumed a pre-post between-group effect size (Cohen *d*) of 0.30 and a dropout rate of 60% at postintervention. To detect the anticipated effect with an 80% probability at a significance level of 5%, a sample size of 586 participants was required.

### Statistical Analyses

All statistical analyses were performed with SPSS Statistics version 27 (IBM Corp). Statistical significance was set at α=.05. Differences in baseline sociodemographic and clinical scores between the intervention group and the control group as well as between participants with poor and good sleep were analyzed using Pearson chi-square tests for dichotomous variables and independent sample *t* tests (2-sided, unpaired) for metric variables. Differences between completers and noncompleters were analyzed in the same way.

Adherence and acceptance data were analyzed descriptively and compared between participants with poor and good sleep by applying independent sample *t* tests. To identify potential predictors of adherence, Pearson correlations were calculated between adherence markers (number of chapters completed and number of sleep diary entries) and baseline variables.

The primary (RIS) and secondary (perceived insomnia-related impairment and PHQ-9 score) outcomes were analyzed using linear repeated-measures mixed-effect models with restricted maximum likelihood estimation and an unstructured covariance matrix. This method follows the intention-to-treat (ITT) approach and is recommended for randomized controlled trials with missing data [85]. Group, time, and interaction of group × time were entered as fixed variables with group as a between-group variable and time as a within-group variable. The outcomes RIS, perceived insomnia-related impairment, and PHQ-9 scores were entered as dependent variables in separate analyses. Within-group effect sizes (Cohen *d*) were calculated by dividing the estimated mean change from baseline to postassessment (or follow-up) by the pooled SD [86]. Between-group effect sizes (Cohen *d*) were computed based on the difference of the estimated mean change from baseline to postassessment (or follow-up) in the intervention group compared with the control group divided by the pooled SD at baseline as recommended by Morris [87].

The analyses for the whole sample were repeated including only participants with poor sleep at baseline as indicated by an RIS score of ≥13 at baseline. Improvements in insomnia symptoms were tested for clinical relevance by comparing the frequency of participants with poor sleep (RIS ≥13) at baseline in both study arms who had improved at postintervention and at follow-up using Pearson chi-square tests for assessment completers. In addition, the odds of suffering from poor sleep (RIS ≥13) at postintervention were compared between the 2 groups using a more conservative ITT approach including all randomized participants with poor sleep. We used a logistic regression model and adjusted for sex, age, and RIS score at baseline. Data on participants who did not complete postassessment questionnaires were imputed by assuming poor sleep at postintervention.

## Results

### Participants

**Figure 1:**
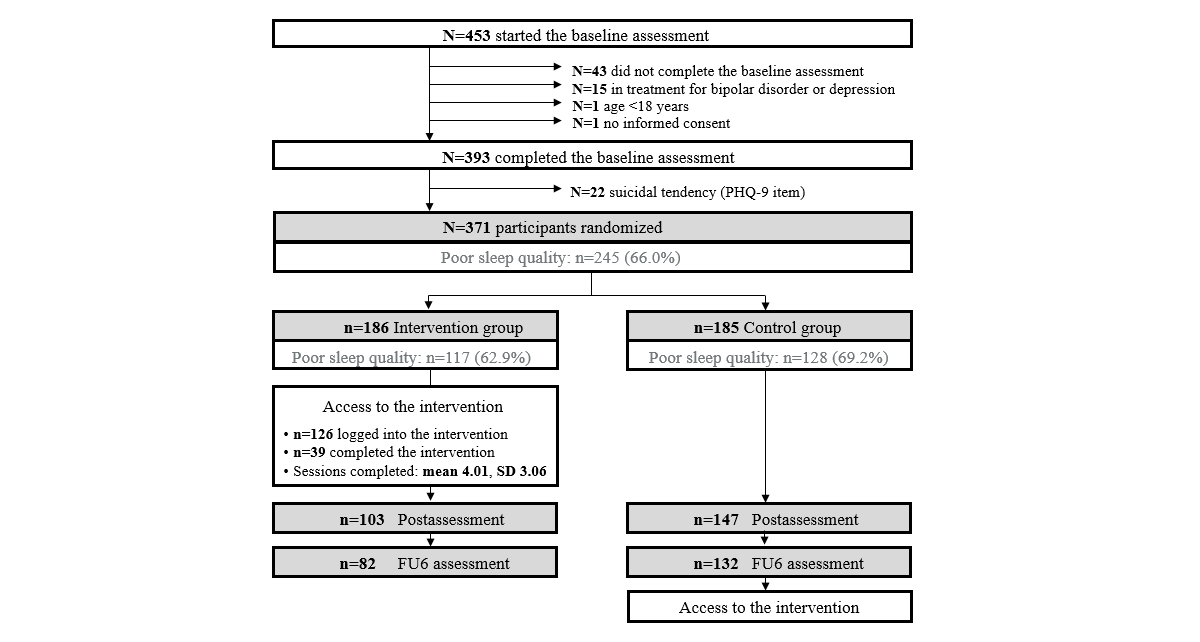
CONSORT (Consolidated Standards of Reporting Trials) flow of participants. FU6: 6-month follow-up; PHQ-9: 9-item Patient Health Questionnaire.

The recruitment period and baseline assessments ran from April 2019 to May 2020. Follow-up data collection was completed in September 2020. Given that the pre-post dropout rate was much smaller than expected, we included fewer participants than originally planned. A total of 393 completed the baseline assessment, of which 371 were randomized (Figure 1). About two-thirds of participants (n=245, 66.0%) reported poor sleep according to an RIS total score of ≥13.

A total of 250 participants (67.4%) provided the primary outcome (RIS total score) at postassessment and 214 (57.7%) completed the RIS at the follow-up assessment. Dropout was higher in the intervention group than in the control group at both assessment points (*χ*^2^_1post_=24.477; *P*<.001 and *χ*^2^_1FU6_=28.247; *P*<.001). Participants with poor initial sleep did not differ from those with good sleep in dropout rates. There also were no differences in any of the baseline measures between study dropouts and completers at postassessment. At follow-up, women (*χ*^2^_1_=5.813; *P*=.02) and participants with a higher level of education (*χ*^2^_1_=7.832; *P*=.005) were more likely to complete the assessment. In the subgroup of participants with poor sleep, education also predicted completion at postassessment (*χ*^2^_1_=5.163; *P*=.02).

The majority of participants in the full sample were women (223/371, 62.8%), in a relationship (271/371, 73.0%), and reported a higher level of education (324/371, 87.3%; Table 2). About 1 in 4 had children and about 1 in 3 were university students. The mean age of participants was 37.3 (SD 14.24) years. Sleep was poor on average (indicated by an RIS score ≥13) and the average level of depression severity was mild.

The subgroups of participants with poor or good sleep differed significantly in most of the sociodemographic variables and in the clinical scores (Table 2). For example, participants with poor sleep were significantly older (*P*<.001), were more likely to have children (*P*<.001), and more likely to be self-employed (*P*=.04). They also reported more severe depressive symptoms (*P*<.001) than those with good sleep and were more likely to receive treatment (*P*<.001) or use medication for their sleep problems (*P*<.001).

There were no significant differences between the intervention group and the control group in sociodemographic characteristics, clinical scores (see Multimedia Appendix 3), or the proportion of participants with poor sleep (117/186, 62.9% vs 128/185, 69.2%, respectively; *χ*^2^_1_=1.634; *P*=.23.

**Table 2:** Baseline characteristics of participants.

Characteristics	Full sample (N=371)	Poor sleep (n=245)	Good sleep (n=126)	*P* value
Female gender, n (%)	233 (62.8)	156 (63.7)	77 (61.1)	.65
In a relationship, n (%)	271 (73.0)	184 (75.1)	87 (69.0)	.23
Children (age <18 years), n (%)	89 (24.0)	69 (28.2)	20 (15.9)	.006
Higher education^a^, n (%)	324 (87.3)	205 (83.7)	119 (94.4)	.003
In education, n (%)	122 (32.9)	60 (24.5)	62 (49.2)	<.001
Self-employed, n (%)	26 (7.0)	22 (9.0)	4 (3.2)	.04
Shift work, n (%)	34 (9.2)	25 (10.2)	9 (7.1)	.33
In treatment, n (%)	41 (11.1)	39 (15.9)	2 (1.6)	<.001
Medication, n (%)	58 (15.6)	58 (23.7)	0 (0.0)	<.001
Sick leave, n (%)	14 (3.8)	13 (5.3)	1 (0.8)	.04
Age, mean (SD)	37.30 (14.24)	41.16 (14.61)	29.82 (9.92)	<.001
RIS^b^ at baseline, mean (SD)	15.33 (6.24)	18.72 (4.63)	8.75 (2.67)	<.001
Perceived insomnia-related impairment, mean (SD)	47.23 (27.37)	58.97 (21.64)	24.40 (22.53)	<.001
PHQ-9^c^ score at baseline, mean (SD)	7.44 (3.56)	8.64 (3.01)	5.11 (3.02)	<.001

^a^At least level 4 according to the European Qualifications Framework.

^b^RIS: Regensburg Insomnia Scale.

^c^PHQ-9: 9-item Patient Health Questionnaire (Depression).

### Adherence to the Intervention

**Figure 2:**
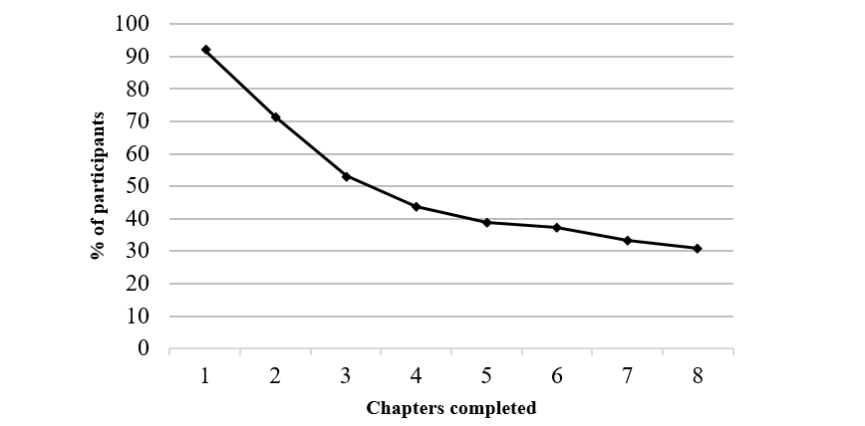
Chapter completion rates in participants who accessed the intervention.

A total of 60/186 (32.3%) participants never logged into the platform or opened the first chapter. Participants who logged into the intervention at least once (n=126) completed on average 4 chapters (SD 3.06; range 0-8) and opened 42/83 (51%) intervention pages (SD 38.14). A total of 39/126 (31.0%) participants completed all chapters of the intervention and 55/126 (43.7%) completed at least half of the intervention; 116/126 (92.1%) completed at least chapter 1 (Figure 2). A total of 106/126 participants (84.1%) used the sleep diary at least once and made entries for 25.23 days (SD 22.3; range 0-83).

Male participants were more likely to not log into the intervention at all (30/65, 46.2% vs 30/121, 24.8%; *χ*^2^_1_=8.829; *P*=.003). Participants with children made fewer entries in the sleep diary than those without (*P*=.02). None of the other sociodemographic variables were predictive for the number of chapters completed or the number of sleep diary entries, and also both RIS total score and PHQ-9 total score at baseline did not predict adherence to the intervention.

### Working Alliance and Acceptance

As many as 87 participants from the intervention group provided WAI-SR data that allowed calculating the score of the Task and Goal subscales. The participants agreed between “sometimes” and “fairly often” with the tasks of the intervention and between “fairly often” and “often” with the goals of the intervention. Acceptance did not differ between participants with poor and good sleep at baseline (Table 3).

Participants who answered the session rating of the intervention chapters rated chapters 1-6 as “good” on average, whereas chapters 7 and 8 were rated as “moderate.” There was no significant difference in session ratings between participants with poor and good sleep, except for session 2 that was rated better and nearly “excellent” by participants with good sleep (*P*<.001).

**Table 3:** Perceived working alliance (WAI-SR^a^ subscales).

Subscale	IG^b^ (all; n=87), mean (SD)	IG poor sleep (n=58), mean (SD)	IG good sleep (n=29), mean (SD)	*P* value
WAI-SR Task	2.78 (0.99)	2.82 (0.96)	2.70 (1.05)	.60
WAI-SR Goal	3.17 (0.98)	3.18 (0.95)	3.16 (1.07)	.95

^a^WAI-SR: Working Alliance Inventory-Short Revised.

^b^IG: intervention group.

### Intervention Effects

Results of the mixed model analyses are presented in Table 4. For the full sample, a significant group × time interaction was found for the RIS at postassessment (*F*_1,269.31_=11.93; *P*=.001) with a small between-group effect (*d*=0.26). Participants in the intervention group showed a stronger reduction (mean difference = –2.66; *d*_within_=0.42) in the RIS scores than those in the control group (mean difference = –1.04; *d*_within_=.17). At follow-up, the group × time interaction failed to reach significance (*F*_1,227.63_=3.54; *P*=.06). Within-group improvements in the perceived insomnia-related impairment due to sleep problems as well as in the PHQ-9 did not differ significantly between the study arms (Table 3).

In the subgroup analyses including only participants with poor sleep at baseline, a significant group × time interaction was found for the RIS at postassessment (*F*_1,180.66_=12.59; *P*<.001; *d*=0.47) and for the perceived insomnia-related impairment at postassessment (*F*_1,184.45_=6.69; *P*=.01; *d*=0.45) as well as for the RIS at the follow-up assessment (*F*_1,147.56_=4.60; *P*=.03; *d*=0.33). Although the intervention group showed somewhat larger reductions in the PHQ-9 than the control group, the difference was not significant (*P*_post_=.13; *P*_FU6_=.15; also see Table 4).

**Table 4:** Results of the intention-to-treat analyses including all participants (N=371) and participants with poor baseline sleep only (n=245).

Results				Baseline, mean (SD)		Post (estimated), mean (SE)		Effect pre-post (within)				Effect pre-post (between)					Follow-up (estimated), mean (SE)			Effect pre-FU6^a^ (within)					Effect pre-FU6 (between)		
								Cohen *d*		95% CI		Cohen *d*		*P* value					Cohen *d*			95% CI		Cohen *d*			*P* value
**All participants (N=371)**																											
	**RIS^b^ total**																										
		IG^c^	15.11 (6.34)		12.45 (0.47)		0.42		0.27 to 0.57		0.26 (0.05 to 0.46)^d^		.001		12.23 (0.52)			0.45			0.30 to 0.60		0.16 (–0.05 to 0.36)			.06	
		CG^e^	15.55 (6.14)		14.51 (0.44)		0.17		0.02 to 0.31		N/A^f^				13.66 (0.46)			0.31			0.16 to 0.45		N/A				
	**Perceived insomnia-related impairment^g^**																										
		IG	45.66 (27.52)		34.76 (2.54)		0.39		0.25 to 0.54		0.17 (–0.04 to 0.37)		.16		35.85 (2.78)			0.36			0.21 to 0.50		0.05 (–0.16 to 0.25)			.67	
		CG	48.81 (27.20)		42.45 (2.22)		0.23		0.09 to 0.37		N/A				40.31 (2.32)			0.31			0.16 to 0.46		N/A				
	**PHQ-9^h^**																										
		IG	7.41 (3.41)		6.13 (0.32)		0.37		0.23 to 0.60		0.13 (–0.08 to 0.33)		.25		6.24 (0.45)			0.34			0.19 to 0.49		0.17 (–0.03 to 0.37)			.25	
		CG	7.47 (3.72)		6.65 (0.30)		0.22		0.07 to 0.37		N/A				6.91 (0.36)			0.15			0.01 to 0.29		N/A				
**Subgroup with poor sleep at baseline (N=245)**																											
	**RIS total**																										
		IG	18.73 (5.02)		15.00 (0.51)		0.74		0.53 to 0.94		0.47 (0.22 to 0.73)^d^		<.001		14.81 (0.62)			0.78			0.57 to 0.98		0.33 (0.08 to 0.59)^d^			.03	
		CG	18.71 (4.27)		17.18 (0.44)		0.36		0.18 to 0.53		N/A				16.34 (0.51)			0.55			0.37 to 0.74		N/A				
	**Perceived insomnia-related impairment**																										
		IG	57.97 (22.17)		41.37 (2.89)		0.74		0.54 to 0.95		0.45 (0.20 to 0.71)^d^		.01		43.27 (3.36)			0.66			0.46 to 0.86		0.26 (0.01 to 0.51)			.18	
		CG	59.88 (21.19)		53.13 (2.43)		0.32		0.14 to 0.49		N/A				50.88 (2.64)			0.42			0.24 to 0.60		N/A				
	**PHQ-9**																										
		IG	8.61 (3.07)		6.94 (0.43)		0.54		0.35 to 0.73		0.25 (0.00 to 0.51)		.13		7.27 (0.60)			0.43			0.24 to 0.62		0.33 (0.08 to 0.59)			.15	
		CG	8.66 (3.34)		7.81 (0.36)		0.25		0.08 to 0.43		N/A				8.40 (0.45)			0.08			–0.10 to 0.25		N/A				

^a^FU6: 6-month follow-up

^b^RIS: Regensburg Insomnia Scale.

^c^IG: intervention group.

^d^Significant group × time interaction.

^e^CG: control group.

^f^N/A: not applicable.

^g^Refers to subjective impairment in the last 7 days (scale ranging from 0 to 100).

^h^PHQ-9: 9-item Patient Health Questionnaire-9 (Depression).

### Evaluation of Clinical Relevance

To assess clinically relevant improvements, we calculated how many participants with poor sleep at baseline had reduced their RIS score below 13 points at postintervention and follow-up assessments. At the postintervention assessment, this was true for 25/69 (36%) assessment completers in the intervention group and 16/102 (15.7%) assessment completers in the control group. This difference was statistically significant (*χ*^2^_1_=9.531; *P*=.002). At follow-up, there was no significant difference between the groups (21/53 IG completers vs 25/90 CG completers; *χ*^2^_1_=2.145; *P*=.14). In the ITT analysis of clinically relevant improvements, the odds of suffering from poor sleep at postintervention were also significantly larger in the control group than in the intervention group (odds ratio 0.462, 95% CI 0.218-0.976; *P*=.02).

To assess clinically relevant deterioration, we assessed how many participants with good sleep at baseline had increased their RIS score to 13 points or more at postintervention and follow-up assessments. At postassessment, this was true for 1/34 (3%) assessment completers in the intervention group and 10/45 (22%) assessment completers in the control group. This difference was significant (*χ*^2^_1_=6.007; *P*=.01). At follow-up, there was no significant difference between the groups (2/29 IG completers vs 2/42 CG completers; *χ*^2^_1_=0.147; *P*=.71).

## Discussion

### Summary of the Trial Objectives

The aim of this study was to evaluate an unguided app-based training to improve sleep quality (insomnia symptoms) in individuals who wish to improve their sleep compared with a waitlist control group. Specifically, we examined participant adherence to the intervention; working alliance and intervention acceptance; and intervention effects on sleep quality (insomnia symptoms), insomnia-related impairment, and depression symptoms.

### Adherence, Working Alliance, and Acceptance

Nonusage attrition was comparable to other digital interventions [88], with a larger loss of participants during the early intervention phase and about 1 in 3 participants completing the intervention. About 1 in 3 participants who were allocated to the intervention group never accessed the intervention. Failure to commence the intervention was 2 times as likely in men, with 1 in 2 never accessing the intervention. The proportion of participants who never started the intervention was markedly lower in a previous study investigating an unguided mobile CBT-I–based intervention [65]. However, we applied broader inclusion criteria compared with [65], that is, we also included individuals reporting slight insomnia symptoms or symptoms below the cutoff (RIS <13). Because our sample was healthier, it can be assumed that the insomnia-related distress of these participants was lower and may have resulted in a lower motivation to start the intervention after completing the baseline assessment. Furthermore, we did not exclude participants with depression. Evidence suggests that adherence to an internet-based CBT-I program is reduced if participants experience other psychiatric or medical problems next to insomnia [89].

Because of the heterogeneity in adherence markers reported (if any were reported), adherence can be compared in detail only with 2 other recent clinical trials [66,90] that investigated unguided digital CBT-I interventions. Participants, on average, completed 4 out of 8 chapters in our study, which is comparable with adherence in another trial, in which participants completed 3.4 out of 6 modules [90]. However, in Lorenz et al [66], adherence was higher, with participants completing 5.6 out of 6 treatment sessions. In our study participants completed the sleep diary, on average, on 25 out of 56 days (45%), whereas they did so for 86% of days in one of the other trials [66].

Participants in the intervention group were able to form a moderate working alliance with the unguided intervention, with a slightly larger concordance regarding goals than regarding tasks. Working alliance in psychotherapy tends to be stronger, but a similar pattern in concordance regarding goals versus tasks is typically also seen in patients undergoing psychotherapy [91], which could be explained by a lesser degree of ambivalence toward treatment goals compared with the actual behavior changes (tasks) that are necessary to achieve them. Working alliance has not been investigated in recent clinical trials investigating unguided digital CBT-I interventions.

The first 6 chapters of the intervention, which provided psychoeducation and various strategies to improve sleep-related habits and behaviors, were on average rated “good,” whereas the final 2 chapters, which mainly focused on reviewing and reinforcing the sleep restriction method and provided fewer new strategies, were rated “moderate.”

### Intervention Effects

#### Sleep Quality (Insomnia Symptoms)

Participants in both groups were able to improve insomnia symptoms during the 6-month assessment period, but participants in the intervention group achieved this improvement faster. In the full sample, the intervention was associated with a statistically significant improvement of insomnia symptoms at postintervention that was maintained at follow-up, although the latter just failed to reach statistical significance. Effect sizes were, however, small. In the subsample of participants with poor sleep quality at baseline, the intervention yielded a medium effect on insomnia symptoms at postintervention and a small effect at follow-up, both being statistically significant. The subgroup effect on insomnia symptoms at postintervention is comparable to that achieved in a previous study evaluating an app-based CBT-I program that did not cover all CBT-I components, with stricter inclusion criteria regarding the presence of insomnia symptoms [65]. In another study that assessed the effects of an app-based CBT-I program, insufficient data have been reported to determine ITT effect sizes, and therefore, a direct comparison is not feasible [71]. Overall, unguided web-based interventions yielded medium to large effects in previous studies [66,68,90,92,93]. Assessment completers with poor sleep at baseline were more likely to achieve clinically relevant improvement at postintervention in the intervention group than in the waitlist group. Moreover, considering all included participants, the odds of suffering from poor sleep at postintervention were higher in the waitlist group than in the intervention group.

In the intervention group, insomnia symptoms improved during the intervention period and then remained unchanged between the postintervention and follow-up periods, whereas in the waitlist group a slight improvement was noted between each of the 3 assessment points. Similar to the intervention group, the improvement in the waitlist group was more pronounced in individuals with poor sleep at baseline. Gradual improvement in insomnia symptoms and sleep quality or efficiency in the untreated control group was also present in some of the other studies on digital CBT-I [65,68,90,92]. These findings indicate that while spontaneous improvements may play a role in insomnia, interventions have the potential of bringing on improvements much faster, thus reducing time with impaired quality of life by promptly supporting individuals at the nadir of their sleep problems.

Our findings also indicate that the intervention may have a protective effect on sleep in individuals with good sleep at baseline. At postintervention, sleep had deteriorated from good to poor only for 1 individual in the intervention group, but over 1 in 5 in the waitlist group. However, that protective effect was not maintained at follow-up.

In comparison with other digital CBT-I studies, we did we did not exclude individuals with insomnia symptoms below the threshold as these individuals still may experience psychological distress or the subjective need to improve their sleep. For these individuals the intervention may have the potential to reduce subthreshold symptoms or prevent symptom progression and the onset of a full syndrome insomnia.

#### Insomnia-Related Impairment

We observed an intervention effect on insomnia-related impairment only in individuals with poor sleep at baseline, which was statistically significant at postintervention, but just failed statistical significance at follow-up. The pattern of improvement in the 2 groups was parallel to that for insomnia symptoms. In the intervention group, the average impairment was reduced during the intervention period and then remained largely unchanged between the postintervention and follow-up periods, whereas in the waitlist group we observed a slight reduction between each of the 3 assessment points. Again, spontaneous improvements in sleep may play a role.

#### Depression

Contrary to other studies on digital CBI-I [65,66,71,90,93], we observed no significant intervention effects on depression. On average, participants in the full sample as well as those in the subsample with poor sleep at baseline on average only reported mild symptoms of depression, so there was limited room for improvement.

### Strengths and Limitations

Compared with previous studies on digital CBT-I–based interventions, we were able to recruit a sample of participants that was large enough to detect even the small intervention effects that are to be expected in an unguided intervention. The study had a waitlist control condition and a longer follow-up period than 1 of the 2 previous studies investigating a mobile intervention [65]. Other than free access to the intervention, study participants received no incentives for taking part in the study or engaging with the intervention, thus avoiding potential incentive-caused bias that may impact intervention adherence. Thus, we can assume that people who used the intervention intensively had an intrinsic motivation to do so. Despite the lack of incentives, dropout at postintervention (121/371, 32.6%) was smaller than expected from our pilot study and within the range of dropout rates (0%-44%) reported in self-help CBT-I interventions [94]. However, not completing trial assessments (ie, dropout attrition [88]) does not necessarily mean that participants do not use an intervention (ie, nonusage attrition). Some people may be interested in using the intervention, but may not wish to complete assessments. Adherence to the intervention was comparable to other trials on digital interventions, and 1 in 3 participants completed the treatment. However, a substantial number of participants never started the intervention. Prerequisites and time requirements to participate in our study were very low. Although many clinical studies require participants to undergo a longer clinical interview during the screening process, participants in our study only had to fill in a relatively short anonymous online survey. This may have resulted in a larger proportion of participants who were ambivalent about the intervention. Although people could participate in the study regardless of whether they had insomnia symptoms, the proportion of individuals with poor sleep at baseline was high, indicating that the intervention was mostly reaching the users it was designed for.

Limitations of our study were the lack of more specific measures for insomnia-related impairment and socioeconomic consequences, including absenteeism or loss of productivity, as well as an objective measure of sleep quality. However, subjective measures of sleep quality and insomnia symptoms are widely used in CBT-I research and the added value of objective measures is a matter of debate [95]. The participants in this trial might have also suffered from sleep disorders other than insomnia (eg, obstructive sleep apnea, circadian rhythm sleep-wake disorders), in which an intervention covering the principles of CBT-I is likely to have limited or no effect. Future studies may thus benefit from including insomnia diagnosis confirmed by a clinician. Adherence to the intervention was approximated by examining usage data. We did not collect information on whether and how participants in the intervention group implemented the suggested behavior changes. Besides, the study design was not suitable to determine the extent to which the components of the interventions contributed to the effects. Secondary analyses are therefore needed to investigate possible moderators or mediators, which are not the subject of this publication. In accordance with other trials evaluating fully automated internet-based CBT-I, the majority of our sample were highly educated participants who are not representative of the general population. However, even if low education is associated with insomnia-related symptoms [96,97], sleep problems are also frequent in people with a higher level of education. Nevertheless, future research should include a more heterogeneous sample with regard to the socioeconomic level and increase efforts to also reach a lower educated population that might be especially underserved.

### Conclusions

In our study, the unguided app-based intervention was associated with a short-term improvement of insomnia symptoms that remained stable over 6 months. Participants in the waitlist condition also reported an improvement in their insomnia symptoms, but this improvement took longer. Effects were more pronounced in individuals with low sleep at baseline. We detected no effect on depression symptoms in our sample, which may largely be due to the low average symptom load at baseline. Although the inclusion criteria for our study were broad, our recruitment strategy mostly reached the individuals the intervention was designed for.

Overall, an anonymous unguided intervention is a feasible option to deliver CBT-I–based treatment to a large number of people, either through self-referral or by primary care providers. Even if the individual effects are smaller than in guided self-help or face-to-face CBT-I, the public health impact could still be profound given the scalability of unguided approaches. Given that very few people have access to face-to-face CBT-I, making it available digitally can increase its availability [98] and public health impact [99]. As in face-to-face CBT-I, some people will not respond to the intervention and precautions must be taken to prevent negative expectations regarding future psychological interventions in those individuals. Symptoms can be continuously measured within the intervention and if participants do not respond, a more intensive treatment can be recommended. Thus, a digital self-help program can serve as a first step of treatment within a stepped care approach [100]. Moderator analyses can contribute to identifying those who will likely benefit from unguided app-based CBT-I and those who need more guidance or face-to-face treatment.

It is noteworthy, however, that 1 in 4 women and 1 in 2 men who were assigned to the intervention group did not start the intervention. In clinical practice, the motivation and ability to adhere to a self-guided intervention should thus be carefully assessed.

In participants who did start the intervention, adherence was comparable to other digital interventions. In future adaptations of the interventions, adherence may be improved by involving a multidisciplinary team of psychologists, user experience specialists, and user experience designers in the design of the user interface, which unfortunately was not possible in our study due to budget constraints. In this context, adherence to the intervention may benefit from persuasive elements such as reminders, automated positive reinforcement, graphical reports, or tailored recommendations. However, such elements need to be implemented in a way that allows users some level of control (eg, options to determine the frequency of reminders [101]). However, adding several persuasive technology features does not necessarily result in better outcomes [102]. Adding some form of human interaction to Refresh could also improve adherence, but this in turn would result in a reduced scalability [103]. Automated feedback that is given when users show low engagement with the intervention may be an alternative [101]. Increased adherence by implementing design features to encourage engagement with the intervention might contribute to more pronounced improvements in insomnia symptoms.

## Appendix

Multimedia Appendix 1 CONSORT-eHEALTH checklist (V 1.6.1). CONSORT: Consolidated Standards of Reporting Trials.

Multimedia Appendix 2 Screenshots of the Refresh intervention.

Multimedia Appendix 3 Baseline characteristics of participants in the intervention group and the control group.

## Abbreviations

CBT-I: cognitive behavioral therapy for insomnia
DSM-5: Diagnostic and Statistical Manual of Mental Disorders, 5th edition
ITT: intention-to-treat
PHQ-9: 9-item Patient Health Questionnaire
REDCap: Research Electronic Data Capture
RIS: Regensburg Insomnia Scale
WAI-SR: Working Alliance Inventory-Short Revised
