# Supplementary material for: Effectiveness of an App-Based Short Intervention to Improve Sleep: Randomized Controlled Trial
Source: JMIR Ment Health. 2023 Mar 21;10:e39052. doi: 10.2196/39052 (PMC10131838; doi:10.2196/39052)
Supplement: Multimedia Appendix 1 [file mental_v10i1e39052_app1.pdf]

CONSORT-EHEALTH (V 1.6.1) - Submission/Publication Form

The CONSORT-EHEALTH checklist is intended for authors of randomized trials evaluating web-based and Internet-based applications/interventions, including mobile interventions, electronic games (incl multiplayer games), social media, certain telehealth applications, and other interactive and/or networked electronic applications. Some of the items (e.g. all subitems under item 5 - description of the intervention) may also be applicable for other study designs.

The goal of the CONSORT EHEALTH checklist and guideline is to be
a) a guide for reporting for authors of RCTs,
b) to form a basis for appraisal of an ehealth trial (in terms of validity)

CONSORT-EHEALTH items/subitems are MANDATORY reporting items for studies published in the Journal of Medical Internet Research and other journals / scientific societies endorsing the checklist.

Items numbered 1., 2., 3., 4a., 4b etc are original CONSORT or CONSORT-NPT (non-pharmacologic treatment) items.
Items with Roman numerals (i., ii, iii, iv etc.) are CONSORT-EHEALTH extensions/clarifications.

As the CONSORT-EHEALTH checklist is still considered in a formative stage, we would ask that you also RATE ON A SCALE OF 1-5 how important/useful you feel each item is FOR THE PURPOSE OF THE CHECKLIST and reporting guideline (optional).

Mandatory reporting items are marked with a red *.
In the textboxes, either copy & paste the relevant sections from your manuscript into this form - please include any quotes from your manuscript in QUOTATION MARKS,
or answer directly by providing additional information not in the manuscript, or elaborating on why the item was not relevant for this study.

YOUR ANSWERS WILL BE PUBLISHED AS A SUPPLEMENTARY FILE TO YOUR PUBLICATION IN JMIR AND ARE CONSIDERED PART OF YOUR PUBLICATION (IF ACCEPTED).
Please fill in these questions diligently. Information will not be copyedited, so please use proper spelling and grammar, use correct capitalization, and avoid abbreviations.

DO NOT FORGET TO SAVE AS PDF _AND_ CLICK THE SUBMIT BUTTON SO YOUR ANSWERS ARE IN OUR DATABASE !!!

Citation Suggestion (if you append the pdf as Appendix we suggest to cite this paper in the caption):
Eysenbach G, CONSORT-EHEALTH Group
CONSORT-EHEALTH: Improving and Standardizing Evaluation Reports of Web-based and Mobile Health Interventions
J Med Internet Res 2011;13(4):e126

URL: http://www.jmir.org/2011/4/e126/
doi: 10.2196/jmir.1923
PMID: 22209829

bianka.vollert@tu-dresden.de wird nicht geteilt Konto wechseln

Entwurf gespeichert

* Erforderlich

Your name *

First Last

Bianka Vollert

Primary Affiliation (short), City, Country *

University of Toronto, Toronto, Canada

TU Dresden, Dresden, Germany

Your e-mail address *

abc@gmail.com

bianka.vollert@tu-dresden.de

Title of your manuscript *

Provide the (draft) title of your manuscript.

Effectiveness of an app-based short intervention to improve sleep: Randomized Controlled Trial

Name of your App/Software/Intervention *

If there is a short and a long/alternate name, write the short name first and add the long name in brackets.

Refresh

Evaluated Version (if any)

e.g. "V1", "Release 2017-03-01", "Version 2.0.27913"

Meine Antwort

Language(s) *

What language is the intervention/app in? If multiple languages are available, separate by comma (e.g. "English, French")

German

URL of your Intervention Website or App

e.g. a direct link to the mobile app on app in appstore (itunes, Google Play), or URL of the website. If the intervention is a DVD or hardware, you can also link to an Amazon page.

Meine Antwort

URL of an image/screenshot (optional)

Meine Antwort

Accessibility *

Can an enduser access the intervention presently?

- ◯ access is free and open
- ◯ access only for special usergroups, not open
- ◯ access is open to everyone, but requires payment/subscription/in-app purchases
- ◉ app/intervention no longer accessible
- ◯ Sonstiges:

Primary Medical Indication/Disease/Condition *

e.g. "Stress", "Diabetes", or define the target group in brackets after the condition, e.g. "Autism (Parents of children with)", "Alzheimers (Informal Caregivers of)"

Insomnia

Primary Outcomes measured in trial *

comma-separated list of primary outcomes reported in the trial

Sleep quality (insomnia symptoms)

Secondary/other outcomes

Are there any other outcomes the intervention is expected to affect?

Insomnia-related impairment, depression

Recommended "Dose" *

What do the instructions for users say on how often the app should be used?

- ○ Approximately Daily
- ◉ Approximately Weekly
- ○ Approximately Monthly
- ○ Approximately Yearly
- ○ "as needed"
- ○ Sonstiges:

Approx. Percentage of Users (starters) still using the app as recommended after 3 months *

- ◉ unknown / not evaluated
- ○ 0-10%
- ○ 11-20%
- ○ 21-30%
- ○ 31-40%
- ○ 41-50%
- ○ 51-60%
- ○ 61-70%
- ○ 71%-80%
- ○ 81-90%
- ○ 91-100%
- ○ Sonstiges:

Overall, was the app/intervention effective? *

- (x) yes: all primary outcomes were significantly better in intervention group vs control
- ( ) partly: SOME primary outcomes were significantly better in intervention group vs control
- ( ) no statistically significant difference between control and intervention
- ( ) potentially harmful: control was significantly better than intervention in one or more outcomes
- ( ) inconclusive: more research is needed
- ( ) Sonstiges:

Article Preparation Status/Stage *

At which stage in your article preparation are you currently (at the time you fill in this form)

- ( ) not submitted yet - in early draft status
- ( ) not submitted yet - in late draft status, just before submission
- ( ) submitted to a journal but not reviewed yet
- (x) submitted to a journal and after receiving initial reviewer comments
- ( ) submitted to a journal and accepted, but not published yet
- ( ) published
- ( ) Sonstiges:

Journal *

If you already know where you will submit this paper (or if it is already submitted), please provide the journal name (if it is not JMIR, provide the journal name under "other")

- ○ not submitted yet / unclear where I will submit this
- ○ Journal of Medical Internet Research (JMIR)
- ○ JMIR mHealth and UHealth
- ○ JMIR Serious Games
- ◉ JMIR Mental Health
- ○ JMIR Public Health
- ○ JMIR Formative Research
- ○ Other JMIR sister journal
- ○ Sonstiges:

Is this a full powered effectiveness trial or a pilot/feasibility trial? *

- ○ Pilot/feasibility
- ◉ Fully powered

Manuscript tracking number *

If this is a JMIR submission, please provide the manuscript tracking number under "other" (The ms tracking number can be found in the submission acknowledgement email, or when you login as author in JMIR. If the paper is already published in JMIR, then the ms tracking number is the four-digit number at the end of the DOI, to be found at the bottom of each published article in JMIR)

- ○ no ms number (yet) / not (yet) submitted to / published in JMIR
- ◉ Sonstiges: JMH ms#39052

TITLE AND ABSTRACT

1a) TITLE: Identification as a randomized trial in the title

1a) Does your paper address CONSORT item 1a? *

I.e does the title contain the phrase "Randomized Controlled Trial"? (if not, explain the reason under "other")

- (selected) yes
- Sonstiges:

1a-i) Identify the mode of delivery in the title

Identify the mode of delivery. Preferably use “web-based” and/or “mobile” and/or “electronic game” in the title. Avoid ambiguous terms like “online”, “virtual”, “interactive”. Use “Internet-based” only if Intervention includes non-web-based Internet components (e.g. email), use “computer-based” or “electronic” only if offline products are used. Use “virtual” only in the context of “virtual reality” (3-D worlds). Use “online” only in the context of “online support groups”. Complement or substitute product names with broader terms for the class of products (such as “mobile” or “smart phone” instead of “iphone”), especially if the application runs on different platforms.

	1	2	3	4	5	
subitem not at all important	○	○	○	○	○	essential

Does your paper address subitem 1a-i? *

Copy and paste relevant sections from manuscript title (include quotes in quotation marks "like this" to indicate direct quotes from your manuscript), or elaborate on this item by providing additional information not in the ms, or briefly explain why the item is not applicable/relevant for your study

"an app-based short intervention"

1a-ii) Non-web-based components or important co-interventions in title

Mention non-web-based components or important co-interventions in title, if any (e.g., “with telephone support”).

	1	2	3	4	5	
subitem not at all important	○	○	○	○	○	essential

Does your paper address subitem 1a-ii?

Copy and paste relevant sections from manuscript title (include quotes in quotation marks "like this" to indicate direct quotes from your manuscript), or elaborate on this item by providing additional information not in the ms, or briefly explain why the item is not applicable/relevant for your study

Meine Antwort

1a-iii) Primary condition or target group in the title

Mention primary condition or target group in the title, if any (e.g., “for children with Type I Diabetes”) Example: A Web-based and Mobile Intervention with Telephone Support for Children with Type I Diabetes: Randomized Controlled Trial

	1	2	3	4	5	
subitem not at all important	○	○	○	○	○	essential

Does your paper address subitem 1a-iii? *

Copy and paste relevant sections from manuscript title (include quotes in quotation marks "like this" to indicate direct quotes from your manuscript), or elaborate on this item by providing additional information not in the ms, or briefly explain why the item is not applicable/relevant for your study

"Effectiveness of an app-based short intervention to improve sleep"

1b) ABSTRACT: Structured summary of trial design, methods, results, and conclusions

NPT extension: Description of experimental treatment, comparator, care providers, centers, and blinding status.

1b-i) Key features/functionalities/components of the intervention and comparator in the METHODS section of the ABSTRACT

Mention key features/functionalities/components of the intervention and comparator in the abstract. If possible, also mention theories and principles used for designing the site. Keep in mind the needs of systematic reviewers and indexers by including important synonyms. (Note: Only report in the abstract what the main paper is reporting. If this information is missing from the main body of text, consider adding it)

	1	2	3	4	5	
subitem not at all important	○	○	○	○	○	essential

Does your paper address subitem 1b-i? *

Copy and paste relevant sections from the manuscript abstract (include quotes in quotation marks "like this" to indicate direct quotes from your manuscript), or elaborate on this item by providing additional information not in the ms, or briefly explain why the item is not applicable/relevant for your study

"In a randomized controlled trial, we evaluated the effects of an app-based short intervention (Refresh) to improve sleep compared to a waitlist condition. Refresh is an eight-week unguided intervention covering the principles of cognitive behavioral therapy for insomnia (CBT-I) and including a sleep diary."

1b-ii) Level of human involvement in the METHODS section of the ABSTRACT

Clarify the level of human involvement in the abstract, e.g., use phrases like “fully automated” vs. “therapist/nurse/care provider/physician-assisted” (mention number and expertise of providers involved, if any). (Note: Only report in the abstract what the main paper is reporting. If this information is missing from the main body of text, consider adding it)

	1	2	3	4	5	
subitem not at all important	○	○	○	○	○	essential

Does your paper address subitem 1b-ii?

Copy and paste relevant sections from the manuscript abstract (include quotes in quotation marks "like this" to indicate direct quotes from your manuscript), or elaborate on this item by providing additional information not in the ms, or briefly explain why the item is not applicable/relevant for your study

"Refresh is an eight-week unguided intervention"

1b-iii) Open vs. closed, web-based (self-assessment) vs. face-to-face assessments in the METHODS section of the ABSTRACT

Mention how participants were recruited (online vs. offline), e.g., from an open access website or from a clinic or a closed online user group (closed usergroup trial), and clarify if this was a purely web-based trial, or there were face-to-face components (as part of the intervention or for assessment). Clearly say if outcomes were self-assessed through questionnaires (as common in web-based trials). Note: In traditional offline trials, an open trial (open-label trial) is a type of clinical trial in which both the researchers and participants know which treatment is being administered. To avoid confusion, use “blinded” or “unblinded” to indicated the level of blinding instead of “open”, as “open” in web-based trials usually refers to “open access” (i.e. participants can self-enrol). (Note: Only report in the abstract what the main paper is reporting. If this information is missing from the main body of text, consider adding it)

	1	2	3	4	5	
subitem not at all important	○	○	○	○	○	essential

Does your paper address subitem 1b-iii?

Copy and paste relevant sections from the manuscript abstract (include quotes in quotation marks "like this" to indicate direct quotes from your manuscript), or elaborate on this item by providing additional information not in the ms, or briefly explain why the item is not applicable/relevant for your study

Outcomes were self-assessed through questionnaires.

1b-iv) RESULTS section in abstract must contain use data

Report number of participants enrolled/assessed in each group, the use/uptake of the intervention (e.g., attrition/adherence metrics, use over time, number of logins etc.), in addition to primary/secondary outcomes. (Note: Only report in the abstract what the main paper is reporting. If this information is missing from the main body of text, consider adding it)

	1	2	3	4	5	
subitem not at all important	○	○	○	○	○	essential

Does your paper address subitem 1b-iv?

Copy and paste relevant sections from the manuscript abstract (include quotes in quotation marks "like this" to indicate direct quotes from your manuscript), or elaborate on this item by providing additional information not in the ms, or briefly explain why the item is not applicable/relevant for your study

"We included 371 participants, of which 245 reported poor sleep at baseline. About one in three participants who were allocated to the intervention group never accessed the intervention. Active participants completed on average four out of eight chapters. Retention rates were 67% (n=250) at post-intervention and 56% (n=214) at six-month follow-up. At post-intervention, insomnia symptoms in the intervention group had improved more than in the waitlist group, with a small effect (d=.26) in the whole sample and a medium effect (d=.45) in the subgroup with poor sleep. Effects in the intervention were maintained at follow-up, while in the waitlist group insomnia symptoms continued to improve during the follow-up period. Perceived insomnia-related impairment also improved from pre- to post-assessment. No significant intervention effect on depression was detected. Working alliance and acceptance were moderate to good."

1b-v) CONCLUSIONS/DISCUSSION in abstract for negative trials

Conclusions/Discussions in abstract for negative trials: Discuss the primary outcome - if the trial is negative (primary outcome not changed), and the intervention was not used, discuss whether negative results are attributable to lack of uptake and discuss reasons. (Note: Only report in the abstract what the main paper is reporting. If this information is missing from the main body of text, consider adding it)

	1	2	3	4	5	
subitem not at all important	○	○	○	○	○	essential

Does your paper address subitem 1b-v?

Copy and paste relevant sections from the manuscript abstract (include quotes in quotation marks "like this" to indicate direct quotes from your manuscript), or elaborate on this item by providing additional information not in the ms, or briefly explain why the item is not applicable/relevant for your study

Meine Antwort

INTRODUCTION

2a) In INTRODUCTION: Scientific background and explanation of rationale

2a-i) Problem and the type of system/solution

Describe the problem and the type of system/solution that is object of the study: intended as stand-alone intervention vs. incorporated in broader health care program? Intended for a particular patient population? Goals of the intervention, e.g., being more cost-effective to other interventions, replace or complement other solutions? (Note: Details about the intervention are provided in "Methods" under 5)

	1	2	3	4	5	
subitem not at all important	○	○	○	○	○	essential

Does your paper address subitem 2a-i? *

Copy and paste relevant sections from the manuscript (include quotes in quotation marks "like this" to indicate direct quotes from your manuscript), or elaborate on this item by providing additional information not in the ms, or briefly explain why the item is not applicable/relevant for your study

"However, reach and access to face-to-face, therapist-delivered CBT-I is limited [41-43], due to both structural and attitudinal barriers. Structural barriers include limited treatment availability, inadequate screening and referrals, and long waiting times [41]. Within-person attitudinal barriers include reservations about help-seeking in general or non pharmacological treatment options in particular [44, 45].
CBT-based self-help interventions may be a means to overcome these barriers and facilitate access to adequate treatment scalable and low-threshold [46]. Especially if they are provided online (Wickwire, 2019). Internet-based interventions have become increasingly popular in the prevention and treatment of mental disorders [e.g., 47], and offer easy accessibility, flexibility, and anonymity. They allow users to integrate an intervention into daily life and in many cases also to automatically monitor progress [48].
There is a growing body of evidence for internet-based CBI¬ I interventions [49, 50].
Meta analyses revealed large improvements in insomnia severity (g = 1.09) and other sleep outcomes (e.g., sleep efficiency and total sleep time) which are comparable to those found in face-to-to interventions [51, 52]. Also, quality of life, executive functions and work-related health and productivity have shown to be improved through internet-based CBT-I [53-56].
Findings indicate that internet-based CBT-I also prevents or reduces a symptoms of depression [57-60].
Fully automated internet-based self-help interventions without human guidance may have the greatest potential when it comes to scalability and cost-effectiveness. While some studies suggest that guided interventions are more effective than unguided ones [52, 61], unguided interventions may still yield large and sustainable effects compared with a waitlist or active control condition [62-67]. "

2a-ii) Scientific background, rationale: What is known about the (type of) system

Scientific background, rationale: What is known about the (type of) system that is the object of the study (be sure to discuss the use of similar systems for other conditions/diagnoses, if appropiate), motivation for the study, i.e. what are the reasons for and what is the context for this specific study, from which stakeholder viewpoint is the study performed, potential impact of findings [2]. Briefly justify the choice of the comparator.

	1	2	3	4	5	
subitem not at all important	○	○	○	○	○	essential

Does your paper address subitem 2a-ii? *

Copy and paste relevant sections from the manuscript (include quotes in quotation marks "like this" to indicate direct quotes from your manuscript), or elaborate on this item by providing additional information not in the ms, or briefly explain why the item is not applicable/relevant for your study

"So far, most research on online interventions has focused on web-based interventions [64]. In the past decade, mobile internet use has been growing and interventions that can be accessed on a smartphone promise a higher potential to reach users than those that require a large screen [68]. On the other hand, mobile use may be associated with different user requirements and expectations, including the length and duration of treatment modules or the presentation of information, which may affect adherence and treatment effects.
While there already is a large number of consumer-targeting apps addressing and tracking sleep (behavior) available in mobile app stores, most of them lack evidence [69]. We only identified two RCTs evaluating app-based interventions for individuals with insomnia. One compared a fully automated Dutch intervention focusing on sleep restriction and relaxation to a waitlist condition [64]. The other compared a Persian self-help intervention based on a combination of Theory of Planned Behavior, Health Action Process Approach and CBT-I to an active control condition (patient education) [70]. While both interventions did not require any human input, the app by [64] included a conversation tool (chatbot) between the app and the participants as well as a number of persuasive strategies. Both RCTs applied strict inclusion criteria for study participation (i.e., insomnia symptoms in accordance with the criteria for a Diagnostic and Statistical Manual of Mental Disorders [DSM-5] diagnosis of insomnia) and found that the app-based intervention was superior to the control condition in the improvement of insomnia symptoms."

2b) In INTRODUCTION: Specific objectives or hypotheses

Does your paper address CONSORT subitem 2b? *

Copy and paste relevant sections from the manuscript (include quotes in quotation marks "like this" to indicate direct quotes from your manuscript), or elaborate on this item by providing additional information not in the ms, or briefly explain why the item is not applicable/relevant for your study

"Given the relative lack of evidence for mobile CBT-I interventions, the aim of the present study is to evaluate an unguided app-based training for individuals who wish to improve their sleep. The primary aim is to evaluate the effect of the intervention on sleep quality (insomnia symptoms). Secondary, exploratory aims include the intervention effects on insomnia-related impairment and depression symptoms as well as participant adherence to the intervention, working alliance and intervention acceptance."

METHODS

3a) Description of trial design (such as parallel, factorial) including allocation ratio

Does your paper address CONSORT subitem 3a? *

Copy and paste relevant sections from the manuscript (include quotes in quotation marks "like this" to indicate direct quotes from your manuscript), or elaborate on this item by providing additional information not in the ms, or briefly explain why the item is not applicable/relevant for your study

"Following an uncontrolled feasibility pilot study with N=189 participants [71] we conducted a randomized controlled trial comparing an intervention group with access to a mobile CBT-I intervention to a waiting list control group. Outcomes were assessed through online questionnaires at baseline (pre-treatment), post-intervention (8 weeks after randomization) and 6-month follow-up."

3b) Important changes to methods after trial commencement (such as eligibility criteria), with reasons

Does your paper address CONSORT subitem 3b? *

Copy and paste relevant sections from the manuscript (include quotes in quotation marks "like this" to indicate direct quotes from your manuscript), or elaborate on this item by providing additional information not in the ms, or briefly explain why the item is not applicable/relevant for your study

This item is not applicable to our study.

3b-i) Bug fixes, Downtimes, Content Changes

Bug fixes, Downtimes, Content Changes: ehealth systems are often dynamic systems. A description of changes to methods therefore also includes important changes made on the intervention or comparator during the trial (e.g., major bug fixes or changes in the functionality or content) (5-iii) and other “unexpected events” that may have influenced study design such as staff changes, system failures/downtimes, etc. [2].

	1	2	3	4	5	
subitem not at all important	○	○	○	○	○	essential

Does your paper address subitem 3b-i?

Copy and paste relevant sections from the manuscript (include quotes in quotation marks "like this" to indicate direct quotes from your manuscript), or elaborate on this item by providing additional information not in the ms, or briefly explain why the item is not applicable/relevant for your study

No changes after trial commercement.

4a) Eligibility criteria for participants

Does your paper address CONSORT subitem 4a? *

Copy and paste relevant sections from the manuscript (include quotes in quotation marks "like this" to indicate direct quotes from your manuscript), or elaborate on this item by providing additional information not in the ms, or briefly explain why the item is not applicable/relevant for your study

"We included participants over 18 years of age who were fluent in German and had access to the Internet during the intervention period. Exclusion criteria were (1) current treatment for depression, (2) a history of psychotic or bipolar disorders and (3) a suicidal ideation according to the answer in the last item of the PHQ-9. Poor sleep was not necessary to participate in the study. All adults showing interest in improving their sleep were welcome if none of the exclusion criteria were present."

4a-i) Computer / Internet literacy

Computer / Internet literacy is often an implicit “de facto” eligibility criterion - this should be explicitly clarified.

	1	2	3	4	5	
subitem not at all important	○	○	○	○	○	essential

Does your paper address subitem 4a-i?

Copy and paste relevant sections from the manuscript (include quotes in quotation marks "like this" to indicate direct quotes from your manuscript), or elaborate on this item by providing additional information not in the ms, or briefly explain why the item is not applicable/relevant for your study

"We included participants over 18 years of age who were fluent in German and had access to the Internet during the intervention period."

4a-ii) Open vs. closed, web-based vs. face-to-face assessments:

Open vs. closed, web-based vs. face-to-face assessments: Mention how participants were recruited (online vs. offline), e.g., from an open access website or from a clinic, and clarify if this was a purely web-based trial, or there were face-to-face components (as part of the intervention or for assessment), i.e., to what degree got the study team to know the participant. In online-only trials, clarify if participants were quasi-anonymous and whether having multiple identities was possible or whether technical or logistical measures (e.g., cookies, email confirmation, phone calls) were used to detect/prevent these.

	1	2	3	4	5	
subitem not at all important	○	○	○	○	○	essential

Does your paper address subitem 4a-ii? *

Copy and paste relevant sections from the manuscript (include quotes in quotation marks "like this" to indicate direct quotes from your manuscript), or elaborate on this item by providing additional information not in the ms, or briefly explain why the item is not applicable/relevant for your study

"Participants were recruited through flyers, postcards and posters in several sleep laboratories in Germany as well as in practices of general practitioners, pharmacies and medical supply stores in Dresden. In addition, recruitment activities took place at Technical University Dresden and included a press release, student email newsletters as well as postcards and posters distributed on the campus. A newspaper article was published in a regional newspaper. Social media channels, e.g., Facebook groups dealing with sleep issues, were used to address further potential participants."

4a-iii) Information giving during recruitment

Information given during recruitment. Specify how participants were briefed for recruitment and in the informed consent procedures (e.g., publish the informed consent documentation as appendix, see also item X26), as this information may have an effect on user self-selection, user expectation and may also bias results.

	1	2	3	4	5	
subitem not at all important	○	○	○	○	○	essential

Does your paper address subitem 4a-iii?

Copy and paste relevant sections from the manuscript (include quotes in quotation marks "like this" to indicate direct quotes from your manuscript), or elaborate on this item by providing additional information not in the ms, or briefly explain why the item is not applicable/relevant for your study

"The recruitment material included a link and a QR-Code leading to the study website. Interested participant received written information about the study and informed consent was obtained online."

4b) Settings and locations where the data were collected

Does your paper address CONSORT subitem 4b? *

Copy and paste relevant sections from the manuscript (include quotes in quotation marks "like this" to indicate direct quotes from your manuscript), or elaborate on this item by providing additional information not in the ms, or briefly explain why the item is not applicable/relevant for your study

"Study data were collected and managed using REDCap electronic data capture tools hosted at the Centre for Clinical Studies at TU Dresden. REDCap [Research Electronic Data Capture; 73] is a secure, web-based application designed to support data capture for research studies, providing: 1) an intuitive interface for validated data entry; 2) audit trails for tracking data manipulation and export procedures; 3) automated export procedures for seamless data downloads to common statistical packages; and 4) procedures for importing data from external sources."

4b-i) Report if outcomes were (self-)assessed through online questionnaires

Clearly report if outcomes were (self-)assessed through online questionnaires (as common in web-based trials) or otherwise.

	1	2	3	4	5	
subitem not at all important	○	○	○	○	○	essential

Does your paper address subitem 4b-i? *

Copy and paste relevant sections from the manuscript (include quotes in quotation marks "like this" to indicate direct quotes from your manuscript), or elaborate on this item by providing additional information not in the ms, or briefly explain why the item is not applicable/relevant for your study

"Outcomes were assessed through online questionnaires at baseline (pre-treatment), post-intervention (8 weeks after randomization) and 6-month follow-up."

4b-ii) Report how institutional affiliations are displayed

Report how institutional affiliations are displayed to potential participants [on ehealth media], as affiliations with prestigious hospitals or universities may affect volunteer rates, use, and reactions with regards to an intervention.(Not a required item – describe only if this may bias results)

	1	2	3	4	5	
subitem not at all important	○	○	○	○	○	essential

Does your paper address subitem 4b-ii?

Copy and paste relevant sections from the manuscript (include quotes in quotation marks "like this" to indicate direct quotes from your manuscript), or elaborate on this item by providing additional information not in the ms, or briefly explain why the item is not applicable/relevant for your study

Meine Antwort

5) The interventions for each group with sufficient details to allow replication, including how and when they were actually administered

5-i) Mention names, credential, affiliations of the developers, sponsors, and owners

Mention names, credential, affiliations of the developers, sponsors, and owners [6] (if authors/evaluators are owners or developer of the software, this needs to be declared in a "Conflict of interest" section or mentioned elsewhere in the manuscript).

	1	2	3	4	5	
subitem not at all important	○	○	○	○	○	essential

Does your paper address subitem 5-i?

Copy and paste relevant sections from the manuscript (include quotes in quotation marks "like this" to indicate direct quotes from your manuscript), or elaborate on this item by providing additional information not in the ms, or briefly explain why the item is not applicable/relevant for your study

Meine Antwort

5-ii) Describe the history/development process

Describe the history/development process of the application and previous formative evaluations (e.g., focus groups, usability testing), as these will have an impact on adoption/use rates and help with interpreting results.

	1	2	3	4	5	
subitem not at all important	○	○	○	○	○	essential

Does your paper address subitem 5-ii?

Copy and paste relevant sections from the manuscript (include quotes in quotation marks "like this" to indicate direct quotes from your manuscript), or elaborate on this item by providing additional information not in the ms, or briefly explain why the item is not applicable/relevant for your study

"Intervention: "Refresh is an unguided app-based intervention adapted and translated from an e-mail delivered CBT for sleep-health program for college students that was originally developed and evaluated in the US [73], and subsequently translated and evaluated in a Japanese college student population [74]. The intervention was adapted for the general adult population and designed as an app-based self-help intervention."

Study Design: "Following an uncontrolled feasibility pilot study with N=189 participants [70] we conducted a randomized controlled trial comparing an intervention group with access to a mobile CBT-I intervention to a waiting list control group."

5-iii) Revisions and updating

Revisions and updating. Clearly mention the date and/or version number of the application/intervention (and comparator, if applicable) evaluated, or describe whether the intervention underwent major changes during the evaluation process, or whether the development and/or content was “frozen” during the trial. Describe dynamic components such as news feeds or changing content which may have an impact on the replicability of the intervention (for unexpected events see item 3b).

	1	2	3	4	5	
subitem not at all important	○	○	○	○	○	essential

Does your paper address subitem 5-iii?

Copy and paste relevant sections from the manuscript (include quotes in quotation marks "like this" to indicate direct quotes from your manuscript), or elaborate on this item by providing additional information not in the ms, or briefly explain why the item is not applicable/relevant for your study

There were no changes during the evaluation process.

5-iv) Quality assurance methods

Provide information on quality assurance methods to ensure accuracy and quality of information provided [1], if applicable.

	1	2	3	4	5	
subitem not at all important	○	○	○	○	○	essential

Does your paper address subitem 5-iv?

Copy and paste relevant sections from the manuscript (include quotes in quotation marks "like this" to indicate direct quotes from your manuscript), or elaborate on this item by providing additional information not in the ms, or briefly explain why the item is not applicable/relevant for your study

Meine Antwort

5-v) Ensure replicability by publishing the source code, and/or providing screenshots/screen-capture video, and/or providing flowcharts of the algorithms used

Ensure replicability by publishing the source code, and/or providing screenshots/screen-capture video, and/or providing flowcharts of the algorithms used. Replicability (i.e., other researchers should in principle be able to replicate the study) is a hallmark of scientific reporting.

	1	2	3	4	5	
subitem not at all important	○	○	○	○	○	essential

Does your paper address subitem 5-v?

Copy and paste relevant sections from the manuscript (include quotes in quotation marks "like this" to indicate direct quotes from your manuscript), or elaborate on this item by providing additional information not in the ms, or briefly explain why the item is not applicable/relevant for your study

Meine Antwort

5-vi) Digital preservation

Digital preservation: Provide the URL of the application, but as the intervention is likely to change or disappear over the course of the years; also make sure the intervention is archived (Internet Archive, webcitation.org, and/or publishing the source code or screenshots/videos alongside the article). As pages behind login screens cannot be archived, consider creating demo pages which are accessible without login.

	1	2	3	4	5	
subitem not at all important	○	○	○	○	○	essential

Does your paper address subitem 5-vi?

Copy and paste relevant sections from the manuscript (include quotes in quotation marks "like this" to indicate direct quotes from your manuscript), or elaborate on this item by providing additional information not in the ms, or briefly explain why the item is not applicable/relevant for your study

This study intervention was accessible only by registered study participants during the study period.

5-vii) Access

Access: Describe how participants accessed the application, in what setting/context, if they had to pay (or were paid) or not, whether they had to be a member of specific group. If known, describe how participants obtained “access to the platform and Internet” [1]. To ensure access for editors/reviewers/readers, consider to provide a “backdoor” login account or demo mode for reviewers/readers to explore the application (also important for archiving purposes, see vi).

	1	2	3	4	5	
subitem not at all important	○	○	○	○	○	essential

Does your paper address subitem 5-vii? *

Copy and paste relevant sections from the manuscript (include quotes in quotation marks "like this" to indicate direct quotes from your manuscript), or elaborate on this item by providing additional information not in the ms, or briefly explain why the item is not applicable/relevant for your study

Participants did not have to be a member of a specific group. The study did not include payments from or to participants.
"Poor sleep was not necessary to participate in the study. All adults showing interest in improving their sleep were welcome if none of the exclusion criteria were present."

5-viii) Mode of delivery, features/functionalities/components of the intervention and comparator, and the theoretical framework

Describe mode of delivery, features/functionalities/components of the intervention and comparator, and the theoretical framework [6] used to design them (instructional strategy [1], behaviour change techniques, persuasive features, etc., see e.g., [7, 8] for terminology). This includes an in-depth description of the content (including where it is coming from and who developed it) [1],” whether [and how] it is tailored to individual circumstances and allows users to track their progress and receive feedback” [6]. This also includes a description of communication delivery channels and – if computer-mediated communication is a component – whether communication was synchronous or asynchronous [6]. It also includes information on presentation strategies [1], including page design principles, average amount of text on pages, presence of hyperlinks to other resources, etc. [1].

	1	2	3	4	5	
subitem not at all important	○	○	○	○	○	essential

Does your paper address subitem 5-viii? *

Copy and paste relevant sections from the manuscript (include quotes in quotation marks "like this" to indicate direct quotes from your manuscript), or elaborate on this item by providing additional information not in the ms, or briefly explain why the item is not applicable/relevant for your study

"The intervention consists of eight consecutive chapters that can be completed in about ten minutes each and cover all CBT-I components recommended by the German Sleep Society (DGSM): Psychoeducation, sleep hygiene, stimulus control, sleep restriction, cognitive restructuring and relaxation [30]. Table 1 summarizes the content of the individual chapters. The chapters are multimedia-based with short text passages, audio and video content, vignettes, as well as questions (multiple choice or free text) to foster an active engagement with the intervention content and also to tailor subsequent content based on the participants' preference (e.g., whether they preferred information to be presented as text or video). The number of pages per chapter and the amount of plain text per page were kept to a minimum as factors like extensive text content and text-content complexity are likely to increase the risk of non-adherence [76]. The intervention is supplemented by a "30-second sleep diary" to be filled in every morning to monitor insomnia symptoms. For a duration of eight weeks, an automated reminder to fill in the sleep diary was sent every morning at 7:00 am. There were no reminders for progressing through the intervention. At the beginning, participants received a short automated feedback about their sleep based on the baseline RIS score. Participants reporting poor sleep were especially encouraged to use parts of the intervention that were marked as for "people with poor sleep" (e.g., sleep restriction)."

5-ix) Describe use parameters

Describe use parameters (e.g., intended "doses" and optimal timing for use). Clarify what instructions or recommendations were given to the user, e.g., regarding timing, frequency, heaviness of use, if any, or was the intervention used ad libitum.

	1	2	3	4	5	
subitem not at all important	○	○	○	○	○	essential

Does your paper address subitem 5-ix?

Copy and paste relevant sections from the manuscript (include quotes in quotation marks "like this" to indicate direct quotes from your manuscript), or elaborate on this item by providing additional information not in the ms, or briefly explain why the item is not applicable/relevant for your study

"The intervention is self-paced, but the recommended duration was eight weeks with one chapter per week."

5-x) Clarify the level of human involvement

Clarify the level of human involvement (care providers or health professionals, also technical assistance) in the e-intervention or as co-intervention (detail number and expertise of professionals involved, if any, as well as “type of assistance offered, the timing and frequency of the support, how it is initiated, and the medium by which the assistance is delivered”. It may be necessary to distinguish between the level of human involvement required for the trial, and the level of human involvement required for a routine application outside of a RCT setting (discuss under item 21 – generalizability).

	1	2	3	4	5	
subitem not at all important	○	○	○	○	○	essential

Does your paper address subitem 5-x?

Copy and paste relevant sections from the manuscript (include quotes in quotation marks "like this" to indicate direct quotes from your manuscript), or elaborate on this item by providing additional information not in the ms, or briefly explain why the item is not applicable/relevant for your study

Refresh is an unguided intervention without any human input or guidance.

5-xi) Report any prompts/reminders used

Report any prompts/reminders used: Clarify if there were prompts (letters, emails, phone calls, SMS) to use the application, what triggered them, frequency etc. It may be necessary to distinguish between the level of prompts/reminders required for the trial, and the level of prompts/reminders for a routine application outside of a RCT setting (discuss under item 21 – generalizability).

	1	2	3	4	5	
subitem not at all important	○	○	○	○	○	essential

Does your paper address subitem 5-xi? *

Copy and paste relevant sections from the manuscript (include quotes in quotation marks "like this" to indicate direct quotes from your manuscript), or elaborate on this item by providing additional information not in the ms, or briefly explain why the item is not applicable/relevant for your study

"Participants in both groups were invited to the post- and follow up assessment via emails. They received up to five reminders at intervals of three days."

"For a duration of eight weeks, an automated reminder to fill in the sleep diary was sent every morning at 7:00 am. There were no reminders for progressing through the intervention."

5-xii) Describe any co-interventions (incl. training/support)

Describe any co-interventions (incl. training/support): Clearly state any interventions that are provided in addition to the targeted eHealth intervention, as ehealth intervention may not be designed as stand-alone intervention. This includes training sessions and support [1]. It may be necessary to distinguish between the level of training required for the trial, and the level of training for a routine application outside of a RCT setting (discuss under item 21 – generalizability.

	1	2	3	4	5	
subitem not at all important	○	○	○	○	○	essential

Does your paper address subitem 5-xii? *

Copy and paste relevant sections from the manuscript (include quotes in quotation marks "like this" to indicate direct quotes from your manuscript), or elaborate on this item by providing additional information not in the ms, or briefly explain why the item is not applicable/relevant for your study

No co-interventions were applied.

6a) Completely defined pre-specified primary and secondary outcome measures, including how and when they were assessed

Does your paper address CONSORT subitem 6a? *

Copy and paste relevant sections from the manuscript (include quotes in quotation marks "like this" to indicate direct quotes from your manuscript), or elaborate on this item by providing additional information not in the ms, or briefly explain why the item is not applicable/relevant for your study

Outcomes were assessed through online questionnaires at baseline (pre-treatment), post-intervention (8 weeks after randomization) and 6-month follow-up. The primary outcome was the change in sleep quality (insomnia symptoms, RIS total score) at post assessment compared to the baseline assessment."

6a-i) Online questionnaires: describe if they were validated for online use and apply CHERRIES items to describe how the questionnaires were designed/deployed

If outcomes were obtained through online questionnaires, describe if they were validated for online use and apply CHERRIES items to describe how the questionnaires were designed/deployed [9].

	1	2	3	4	5	
subitem not at all important	○	○	○	○	○	essential

Does your paper address subitem 6a-i?

Copy and paste relevant sections from manuscript text

"Regensburg Insomnia Scale (RIS). Sleep quality (insomnia symptoms) was assessed using the German Regensburg Insomnia Scale [72]. The scale covers psychophysiological (cognitive, emotional and behavioral) aspects of insomnia during the previous four weeks. The instrument consists of ten items to be answered on a 5-point self-rating scale ranging from 0 to 4. A total score of ≥13 indicates poor sleep [72]. The RIS has shown to be a well accepted, valid instrument that has discriminative power and is sensitive to detect improvements in insomnia parameters after CBT-I [72].
One additional item asked the participants about their perceived insomnia-related impairment in the past seven days on a scale ranging from 0 to 100.
Patient Health Questionnaire-9 (PHQ-9). Depressive symptoms and depression severity were measured using the German version of the Patient Health Questionnaire-9 [77]. This widely used brief self-report instrument consists of nine items covering the DSM-5 diagnostic criteria of depression which are scored on a four-point Likert scale from 0 (“not at all”) to 3 (“nearly every day”). The PHQ-9 has been shown to have good psychometric properties [78] and also has demonstrated to be sensitive to changes in depressive symptomatology over time [79].
Working Alliance Inventory-Short Revised (WAI-SR). Acceptance of the intervention was assessed using the subscales “task” and “goal” of the Working Alliance Inventory-Short Revised [80, 81], adapted for online interventions. The inventory is based on Bordin's alliance theory [82]. The “task” subscale (4 items) measures the agreement on the tasks of the intervention, whereas the „goal“ subscale (4 items) measures the agreement on the goals of the intervention. Due to the unguided nature of the study, the “bond” subscale (measuring the quality of an affective bond between patient [participant] and therapist [coach]) was removed. Items are answered on a scale from 1 (“seldom”) to 5 (“always”), but participants could also choose the additional answer category “I don’t know”. In addition to the WAI-SR, each chapter of the intervention could be rated on a five-point scale."

6a-ii) Describe whether and how “use” (including intensity of use/dosage) was defined/measured/monitored

Describe whether and how “use” (including intensity of use/dosage) was defined/measured/monitored (logins, logfile analysis, etc.). Use/adoption metrics are important process outcomes that should be reported in any ehealth trial.

	1	2	3	4	5	
subitem not at all important	○	○	○	○	○	essential

Does your paper address subitem 6a-ii?

Copy and paste relevant sections from manuscript text

"Adherence to the intervention was defined as the number of chapters completed and the proportional progress through the intervention (in percent). In addition, the number of entries in the sleep diary was used to describe the usage of the intervention."

6a-iii) Describe whether, how, and when qualitative feedback from participants was obtained

Describe whether, how, and when qualitative feedback from participants was obtained (e.g., through emails, feedback forms, interviews, focus groups).

	1	2	3	4	5	
subitem not at all important	○	○	○	○	○	essential

Does your paper address subitem 6a-iii?

Copy and paste relevant sections from manuscript text

Qualitative feedback was not a part of this RCT, but the Working Alliance Inventory-Short Revised (WAI-SR) and session ratings were applied in order to evaluate the acceptance of the intervention.

6b) Any changes to trial outcomes after the trial commenced, with reasons

Does your paper address CONSORT subitem 6b? *

Copy and paste relevant sections from the manuscript (include quotes in quotation marks "like this" to indicate direct quotes from your manuscript), or elaborate on this item by providing additional information not in the ms, or briefly explain why the item is not applicable/relevant for your study

There were no changes required.

7a) How sample size was determined

NPT: When applicable, details of whether and how the clustering by care provides or centers was addressed

7a-i) Describe whether and how expected attrition was taken into account when calculating the sample size

Describe whether and how expected attrition was taken into account when calculating the sample size.

	1	2	3	4	5	
subitem not at all important	○	○	○	○	○	essential

Does your paper address subitem 7a-i?

Copy and paste relevant sections from manuscript title (include quotes in quotation marks "like this" to indicate direct quotes from your manuscript), or elaborate on this item by providing additional information not in the ms, or briefly explain why the item is not applicable/relevant for your study

"Based on results from the pilot study [71], we assumed a pre-post between-group effect size (Cohen's d) of 0.30 and a dropout rate of 60% at post-intervention. To detect the anticipated effect with an 80% probability at a significance level of 5%, a sample size of N=586 participants was required."

7b) When applicable, explanation of any interim analyses and stopping guidelines

Does your paper address CONSORT subitem 7b? *

Copy and paste relevant sections from the manuscript (include quotes in quotation marks "like this" to indicate direct quotes from your manuscript), or elaborate on this item by providing additional information not in the ms, or briefly explain why the item is not applicable/relevant for your study

We closed the recruitment when we had reached the required number of completed post assessments (according to our sample size calculation).
"Given that the pre-post dropout rate was much smaller than expected, we included fewer participants than originally planned."

8a) Method used to generate the random allocation sequence
NPT: When applicable, how care providers were allocated to each trial group

Does your paper address CONSORT subitem 8a? *

Copy and paste relevant sections from the manuscript (include quotes in quotation marks "like this" to indicate direct quotes from your manuscript), or elaborate on this item by providing additional information not in the ms, or briefly explain why the item is not applicable/relevant for your study

Respondents were allocated to either the intervention or control group by means of an automated randomization algorithm implemented in REDCap.

8b) Type of randomisation; details of any restriction (such as blocking and block size)

Does your paper address CONSORT subitem 8b? *

Copy and paste relevant sections from the manuscript (include quotes in quotation marks "like this" to indicate direct quotes from your manuscript), or elaborate on this item by providing additional information not in the ms, or briefly explain why the item is not applicable/relevant for your study

"Participants were randomized in a 1:1 ratio. The randomization was block stratified by gender (female vs. male), severity of insomnia symptoms (poor [RIS total score ≥13 [72]] vs. good sleep [RIS total score <13]) and the consumption of sleep-inducing drugs (yes vs. no)."

9) Mechanism used to implement the random allocation sequence (such as sequentially numbered containers), describing any steps taken to conceal the sequence until interventions were assigned

Does your paper address CONSORT subitem 9? *

Copy and paste relevant sections from the manuscript (include quotes in quotation marks "like this" to indicate direct quotes from your manuscript), or elaborate on this item by providing additional information not in the ms, or briefly explain why the item is not applicable/relevant for your study

Randomization took place after a respondent had completed the baseline questionnaires and was checked for eligibility. A research assistant without access to the implemented random allocation sequence randomized the participants after baseline in REDCap.

10) Who generated the random allocation sequence, who enrolled participants, and who assigned participants to interventions

Does your paper address CONSORT subitem 10? *

Copy and paste relevant sections from the manuscript (include quotes in quotation marks "like this" to indicate direct quotes from your manuscript), or elaborate on this item by providing additional information not in the ms, or briefly explain why the item is not applicable/relevant for your study

The random allocation sequence was implemented on the data collection platform.

11a) If done, who was blinded after assignment to interventions (for example, participants, care providers, those assessing outcomes) and how

NPT: Whether or not administering co-interventions were blinded to group assignment

11a-i) Specify who was blinded, and who wasn’t

Specify who was blinded, and who wasn’t. Usually, in web-based trials it is not possible to blind the participants [1, 3] (this should be clearly acknowledged), but it may be possible to blind outcome assessors, those doing data analysis or those administering co-interventions (if any).

	1	2	3	4	5	
subitem not at all important	○	○	○	○	○	essential

Does your paper address subitem 11a-i? *

Copy and paste relevant sections from the manuscript (include quotes in quotation marks "like this" to indicate direct quotes from your manuscript), or elaborate on this item by providing additional information not in the ms, or briefly explain why the item is not applicable/relevant for your study

Participants were not blinded to the group they were allocated to.

11a-ii) Discuss e.g., whether participants knew which intervention was the “intervention of interest” and which one was the “comparator”

Informed consent procedures (4a-ii) can create biases and certain expectations - discuss e.g., whether participants knew which intervention was the “intervention of interest” and which one was the “comparator”.

	1	2	3	4	5	
subitem not at all important	○	○	○	○	○	essential

Does your paper address subitem 11a-ii?

Copy and paste relevant sections from the manuscript (include quotes in quotation marks "like this" to indicate direct quotes from your manuscript), or elaborate on this item by providing additional information not in the ms, or briefly explain why the item is not applicable/relevant for your study

The intervention group was compared to a control group without access to the intervention during the study period. There was no active control group.

11b) If relevant, description of the similarity of interventions

(this item is usually not relevant for ehealth trials as it refers to similarity of a placebo or sham intervention to a active medication/intervention)

Does your paper address CONSORT subitem 11b? *

Copy and paste relevant sections from the manuscript (include quotes in quotation marks "like this" to indicate direct quotes from your manuscript), or elaborate on this item by providing additional information not in the ms, or briefly explain why the item is not applicable/relevant for your study

Not applicable to our study.

12a) Statistical methods used to compare groups for primary and secondary outcomes

NPT: When applicable, details of whether and how the clustering by care providers or centers was addressed

Does your paper address CONSORT subitem 12a? *

Copy and paste relevant sections from the manuscript (include quotes in quotation marks "like this" to indicate direct quotes from your manuscript), or elaborate on this item by providing additional information not in the ms, or briefly explain why the item is not applicable/relevant for your study

"All statistical analyses were performed with IBM SPSS Statistics version 27. Statistical significance was set at α=.05. Differences in baseline socio-demographic and clinical scores between the intervention and the control group as well as between participants with poor vs. good sleep were analyzed using Pearson χ^2-tests for dichotomous variables and independent-sample t-tests (two-sided) for metric variables. Differences between completers and non-completers were analyzed in the same way.
Adherence and acceptance data were analyzed descriptively and compared between participants with a poor vs. a good sleep applying independent-sample t-tests. To identify potential predictors of adherence, Pearson's correlations were calculated between adherence markers (number of chapters completed, number of sleep diary entries) and baseline variables.
Primary (RIS) and secondary (perceived insomnia-related impairment, PHQ-9) outcomes were analyzed using linear repeated-measures mixed-effect models with restricted maximum likelihood estimation (REML) and an unstructured covariance matrix. This method follows the ITT approach and is recommended for RCTs with missing data [83]. Group, time and interaction of group and time were entered as fixed variables with group as a between-group variable and time as a within-group variable. The outcomes RIS, perceived insomnia-related impairment and PHQ-9 were entered as dependent variables in separate analyses. Within-group effect sizes (Cohen's d) were calculated by dividing the estimated mean change from baseline to post-assessment (or follow-up) by the pooled standard deviation [84]. Between-group effect sizes (Cohen's d) were computed based on the difference of the estimated mean change from baseline to post-assessment (or follow-up) in the intervention group compared to the control group divided by the pooled standard deviation at baseline as recommended by [85]."

12a-i) Imputation techniques to deal with attrition / missing values

Imputation techniques to deal with attrition / missing values: Not all participants will use the intervention/comparator as intended and attrition is typically high in ehealth trials. Specify how participants who did not use the application or dropped out from the trial were treated in the statistical analysis (a complete case analysis is strongly discouraged, and simple imputation techniques such as LOCF may also be problematic [4]).

	1	2	3	4	5	
subitem not at all important	○	○	○	○	○	essential

Does your paper address subitem 12a-i? *

Copy and paste relevant sections from the manuscript (include quotes in quotation marks "like this" to indicate direct quotes from your manuscript), or elaborate on this item by providing additional information not in the ms, or briefly explain why the item is not applicable/relevant for your study

"Primary (RIS) and secondary (perceived insomnia-related impairment, PHQ-9) outcomes were analyzed using linear repeated-measures mixed-effect models with restricted maximum likelihood estimation (REML) and an unstructured covariance matrix. This method follows the ITT approach and is recommended for RCTs with missing data [83]."

"In addition, the odds of suffering from poor sleep (RIS≥13) at post-intervention was compared between the two groups using a more conservative ITT approach including all randomized participants with poor sleep. We used a logistic regression model and adjusted for sex, age, and RIS score at baseline. Data of participants who did not complete post-assessments were imputed assuming poor sleep at post-intervention."

12b) Methods for additional analyses, such as subgroup analyses and adjusted analyses

Does your paper address CONSORT subitem 12b? *

Copy and paste relevant sections from the manuscript (include quotes in quotation marks "like this" to indicate direct quotes from your manuscript), or elaborate on this item by providing additional information not in the ms, or briefly explain why the item is not applicable/relevant for your study

"The analyses for the whole sample were repeated including only participants with poor sleep at baseline as indicated by a RIS score ≥13 at baseline. Improvements in insomnia symptoms were tested for clinical relevance comparing the frequency of participants with poor sleep (RIS≥13) at baseline in both study arms who had improved at post-intervention and follow-up using Pearson χ^2-tests for assessment completers. In addition, the odds of suffering from poor sleep (RIS≥13) at post-intervention was compared between the two groups using a more conservative ITT approach including all randomized participants with poor sleep. We used a logistic regression model and adjusted for sex, age, and RIS score at baseline. Data of participants who did not complete post-assessments were imputed assuming poor sleep at post-intervention."

X26) REB/IRB Approval and Ethical Considerations [recommended as subheading under "Methods"] (not a CONSORT item)

X26-i) Comment on ethics committee approval

	1	2	3	4	5	
subitem not at all important	○	○	○	○	○	essential

Does your paper address subitem X26-i?

Copy and paste relevant sections from the manuscript (include quotes in quotation marks "like this" to indicate direct quotes from your manuscript), or elaborate on this item by providing additional information not in the ms, or briefly explain why the item is not applicable/relevant for your study

"The study was approved by the Ethics committee (IRB) at TU Dresden (ref: EK 111032919)"

x26-ii) Outline informed consent procedures

Outline informed consent procedures e.g., if consent was obtained offline or online (how? Checkbox, etc.?), and what information was provided (see 4a-ii). See [6] for some items to be included in informed consent documents.

	1	2	3	4	5	
subitem not at all important	○	○	○	○	○	essential

Does your paper address subitem X26-ii?

Copy and paste relevant sections from the manuscript (include quotes in quotation marks "like this" to indicate direct quotes from your manuscript), or elaborate on this item by providing additional information not in the ms, or briefly explain why the item is not applicable/relevant for your study

Informed consent was obtained online.

X26-iii) Safety and security procedures

Safety and security procedures, incl. privacy considerations, and any steps taken to reduce the likelihood or detection of harm (e.g., education and training, availability of a hotline)

	1	2	3	4	5	
subitem not at all important	○	○	○	○	○	essential

Does your paper address subitem X26-iii?

Copy and paste relevant sections from the manuscript (include quotes in quotation marks "like this" to indicate direct quotes from your manuscript), or elaborate on this item by providing additional information not in the ms, or briefly explain why the item is not applicable/relevant for your study

In case of exlusion from the study due to suicidal ideation (according to the PHQ-9 item), the respondent received recommendations about support services.

Study data were collected and managed using REDCap electronic data capture tools hosted at the Centre for Clinical Studies at TU Dresden. REDCap [Research Electronic Data Capture; 72] is a secure, web-based application designed to support data capture for research studies

RESULTS

13a) For each group, the numbers of participants who were randomly assigned, received intended treatment, and were analysed for the primary outcome

NPT: The number of care providers or centers performing the intervention in each group and the number of patients treated by each care provider in each center

Does your paper address CONSORT subitem 13a? *

Copy and paste relevant sections from the manuscript (include quotes in quotation marks "like this" to indicate direct quotes from your manuscript), or elaborate on this item by providing additional information not in the ms, or briefly explain why the item is not applicable/relevant for your study

"A total of N=393 completed the baseline assessment, of which N=371 were randomized (see Figure 1). About two thirds of participants (N=245; 66%) reported poor sleep according to a RIS total score of ≥13.
N=250 participants (67.4%) provided the primary outcome (RIS total score) at post assessment and N=214 (57.7%) completed the RIS at follow-up assessment. Dropout was higher in the intervention group than in the control group at both assessment points (χ^2_{post}=24.477; P<.001; χ^2_{FU6}=28.247; p<.001)."

13b) For each group, losses and exclusions after randomisation, together with reasons

Does your paper address CONSORT subitem 13b? (NOTE: Preferably, this is shown in a CONSORT flow diagram) *

Copy and paste relevant sections from the manuscript (include quotes in quotation marks "like this" to indicate direct quotes from your manuscript), or elaborate on this item by providing additional information not in the ms, or briefly explain why the item is not applicable/relevant for your study

"A total of N=393 completed the baseline assessment, of which N=371 were randomized (see Figure 1). About two thirds of participants (N=245; 66%) reported poor sleep according to a RIS total score of ≥13.
N=250 participants (67.4%) provided the primary outcome (RIS total score) at post assessment and N=214 (57.7%) completed the RIS at follow-up assessment. Dropout was higher in the intervention group than in the control group at both assessment points (χ^2post=24.477; P<.001; χ^2FU6=28.247; p<.001). Participants with a poor initial sleep did not differ from those with good sleep in dropout rates."

13b-i) Attrition diagram

Strongly recommended: An attrition diagram (e.g., proportion of participants still logging in or using the intervention/comparator in each group plotted over time, similar to a survival curve) or other figures or tables demonstrating usage/dose/engagement.

	1	2	3	4	5	
subitem not at all important	○	○	○	○	○	essential

Does your paper address subitem 13b-i?

Copy and paste relevant sections from the manuscript or cite the figure number if applicable (include quotes in quotation marks "like this" to indicate direct quotes from your manuscript), or elaborate on this item by providing additional information not in the ms, or briefly explain why the item is not applicable/relevant for your study

"N=60 (32.3%) participants never logged onto the platform or opened the first chapter. Participants who logged onto the intervention at least once (N=126) completed on average four chapters (SD=3.06; range 0-8) and opened 51% of the intervention pages (SD=38.14). N=39 (31.0%) completed all chapters of the intervention and N=55 (43.7%) completed at least half of the intervention. N=116 (92.1%) completed at least chapter 1 (see Figure 2). N=106 participants (84.1%) used the sleep diary at least once and made entries for 25.23 days (SD=22.3, range 0-83)."

14a) Dates defining the periods of recruitment and follow-up

Does your paper address CONSORT subitem 14a? *

Copy and paste relevant sections from the manuscript (include quotes in quotation marks "like this" to indicate direct quotes from your manuscript), or elaborate on this item by providing additional information not in the ms, or briefly explain why the item is not applicable/relevant for your study

"The recruitment period and baseline assessment ran from April 2019 to May 2020. Follow-up data collection was completed in September 2020."

14a-i) Indicate if critical "secular events" fell into the study period

Indicate if critical "secular events" fell into the study period, e.g., significant changes in Internet resources available or "changes in computer hardware or Internet delivery resources"

	1	2	3	4	5	
subitem not at all important	○	○	○	○	○	essential

Does your paper address subitem 14a-i?

Copy and paste relevant sections from the manuscript (include quotes in quotation marks "like this" to indicate direct quotes from your manuscript), or elaborate on this item by providing additional information not in the ms, or briefly explain why the item is not applicable/relevant for your study

Meine Antwort

14b) Why the trial ended or was stopped (early)

Does your paper address CONSORT subitem 14b? *

Copy and paste relevant sections from the manuscript (include quotes in quotation marks "like this" to indicate direct quotes from your manuscript), or elaborate on this item by providing additional information not in the ms, or briefly explain why the item is not applicable/relevant for your study

The trial was ended when the last FU assessment had been completed (approximately 6 months after the last participant was enrolled).

15) A table showing baseline demographic and clinical characteristics for each group

NPT: When applicable, a description of care providers (case volume, qualification, expertise, etc.) and centers (volume) in each group

Does your paper address CONSORT subitem 15? *

Copy and paste relevant sections from the manuscript (include quotes in quotation marks "like this" to indicate direct quotes from your manuscript), or elaborate on this item by providing additional information not in the ms, or briefly explain why the item is not applicable/relevant for your study

Table 2. Baseline characteristics of participants

15-i) Report demographics associated with digital divide issues

In ehealth trials it is particularly important to report demographics associated with digital divide issues, such as age, education, gender, social-economic status, computer/Internet/ehealth literacy of the participants, if known.

	1	2	3	4	5	
subitem not at all important	○	○	○	○	○	essential

Does your paper address subitem 15-i? *

Copy and paste relevant sections from the manuscript (include quotes in quotation marks "like this" to indicate direct quotes from your manuscript), or elaborate on this item by providing additional information not in the ms, or briefly explain why the item is not applicable/relevant for your study

"The majority of participants in the full sample were women (63.7%), in a relationship (73.0%) and reported a higher level of education (87.3%; see Table 2). About one in four had children and about one in three were university students. The mean age of participants was 37.3 years (SD=12.24). Sleep was poor on average (indicated by the RIS score ≥ 13) and the average level of depression severity was mild."

16) For each group, number of participants (denominator) included in each analysis and whether the analysis was by original assigned groups

16-i) Report multiple “denominators” and provide definitions

Report multiple “denominators” and provide definitions: Report N’s (and effect sizes) “across a range of study participation [and use] thresholds” [1], e.g., N exposed, N consented, N used more than x times, N used more than y weeks, N participants “used” the intervention/comparator at specific pre-defined time points of interest (in absolute and relative numbers per group). Always clearly define “use” of the intervention.

	1	2	3	4	5	
subitem not at all important	○	○	○	○	○	essential

Does your paper address subitem 16-i? *

Copy and paste relevant sections from the manuscript (include quotes in quotation marks "like this" to indicate direct quotes from your manuscript), or elaborate on this item by providing additional information not in the ms, or briefly explain why the item is not applicable/relevant for your study

See Figure 1.
"N=60 (32.3%) participants never logged onto the platform or opened the first chapter. Participants who logged onto the intervention at least once (N=126) completed on average four chapters (SD=3.06; range 0-8) and opened 51% of the intervention pages (SD=38.14). N=39 (31.0%) completed all chapters of the intervention and N=55 (43.7%) completed at least half of the intervention."

16-ii) Primary analysis should be intent-to-treat

Primary analysis should be intent-to-treat, secondary analyses could include comparing only "users", with the appropriate caveats that this is no longer a randomized sample (see 18-i).

	1	2	3	4	5	
subitem not at all important	○	○	○	○	○	essential

Does your paper address subitem 16-ii?

Copy and paste relevant sections from the manuscript (include quotes in quotation marks "like this" to indicate direct quotes from your manuscript), or elaborate on this item by providing additional information not in the ms, or briefly explain why the item is not applicable/relevant for your study

"Results of the mixed model analyses are presented in Table 4."

17a) For each primary and secondary outcome, results for each group, and the estimated effect size and its precision (such as 95% confidence interval)

Does your paper address CONSORT subitem 17a? *

Copy and paste relevant sections from the manuscript (include quotes in quotation marks "like this" to indicate direct quotes from your manuscript), or elaborate on this item by providing additional information not in the ms, or briefly explain why the item is not applicable/relevant for your study

"Results of the mixed model analyses are presented in Table 4. For the full sample, a significant group × time interaction was found for the RIS at post assessment (F1, 269.31=11.93; P=.001) with a small between-group effect (d=.26). Participants in the intervention group showed a stronger reduction (mean difference = –2.66; dwithin=.45) in the RIS scores than participants in the control group (mean difference = –1.04; dwithin=.17). At follow up, the group × time interaction failed to reach significance (F1, 227.63=3.54; P=.06). Within-group improvements in the perceived insomnia-related impairment due to sleep problems as well as in the PHQ-9 did not differ significantly between the study arms."

17a-i) Presentation of process outcomes such as metrics of use and intensity of use

In addition to primary/secondary (clinical) outcomes, the presentation of process outcomes such as metrics of use and intensity of use (dose, exposure) and their operational definitions is critical. This does not only refer to metrics of attrition (13-b) (often a binary variable), but also to more continuous exposure metrics such as “average session length”. These must be accompanied by a technical description how a metric like a “session” is defined (e.g., timeout after idle time) [1] (report under item 6a).

	1	2	3	4	5	
subitem not at all important	○	○	○	○	○	essential

Does your paper address subitem 17a-i?

Copy and paste relevant sections from the manuscript (include quotes in quotation marks "like this" to indicate direct quotes from your manuscript), or elaborate on this item by providing additional information not in the ms, or briefly explain why the item is not applicable/relevant for your study

"N=60 (32.3%) participants never logged onto the platform or opened the first chapter. Participants who logged onto the intervention at least once (N=126) completed on average four chapters (SD=3.06; range 0-8) and opened 51% of the intervention pages (SD=38.14). N=39 (31.0%) completed all chapters of the intervention and N=55 (43.7%) completed at least half of the intervention. N=116 (92.1%) completed at least chapter 1 (see Figure 2). N=106 participants (84.1%) used the sleep diary at least once and made entries for 25.23 days (SD=22.3, range 0-83)."
"Complete WAI-SR that allows calculating scores data is available for N=87 participants from the intervention group. The participants agreed between “sometimes” and “fairly often” with the tasks of the intervention and between “fairly often” and “often” with the goals of the intervention. Acceptance did not differ between participants with poor and good sleep at baseline (see Table 3).
Participants who answered the session rating of the intervention chapters rated chapters 1-6 as “good” on average, whereas chapter 7-8 were rated as “moderate”. There was no significant difference in session ratings between participants with poor vs. good sleep, except for session 2 that was rated better and nearly “excellent” by participants with good sleep."

17b) For binary outcomes, presentation of both absolute and relative effect sizes is recommended

Does your paper address CONSORT subitem 17b? *

Copy and paste relevant sections from the manuscript (include quotes in quotation marks "like this" to indicate direct quotes from your manuscript), or elaborate on this item by providing additional information not in the ms, or briefly explain why the item is not applicable/relevant for your study

Not applicable to our paper.

18) Results of any other analyses performed, including subgroup analyses and adjusted analyses, distinguishing pre-specified from exploratory

Does your paper address CONSORT subitem 18? *

Copy and paste relevant sections from the manuscript (include quotes in quotation marks "like this" to indicate direct quotes from your manuscript), or elaborate on this item by providing additional information not in the ms, or briefly explain why the item is not applicable/relevant for your study

"In the subgroup analyses including only participants with poor sleep at baseline, a significant group × time interaction was found for the RIS at post assessment (F1, 180.66=12.59; P=<.001; d=.47) and for the perceived insomnia-related impairment at post assessment (F1, 184.45=6.69; P=.01; d=.45) as well as for the RIS at follow up assessment (F1, 147.56=4.60; P=.03; d=.33). Although the intervention group showed somewhat larger reductions in the PHQ-9 than the control group, the difference was not significant."
"To assess clinically relevant improvements, we assessed how many participants with poor sleep at baseline had reduced their RIS score below 13 points at post-intervention and follow-up. At post-intervention, this was true for N=25 out of 69 (36.2%) assessment completers in the intervention group and N=16 out of 102 (15.7%) assessment completers in the control group. This difference is statistically significant (χ^2=9.531; P=.002). At follow up, there was no significant difference between the groups (21 out of 53 IG completers vs.
25 out of 90 CG completers; χ^2=2.145; P=.143). In the ITT analysis of clinically relevant improvements, the odds of suffering from poor sleep at post-intervention was also significantly larger in the control group than in the intervention group (OR=0.462 [0.218 -0.976])."

18-i) Subgroup analysis of comparing only users

A subgroup analysis of comparing only users is not uncommon in ehealth trials, but if done, it must be stressed that this is a self-selected sample and no longer an unbiased sample from a randomized trial (see 16-iii).

	1	2	3	4	5	
subitem not at all important	○	○	○	○	○	essential

Does your paper address subitem 18-i?

Copy and paste relevant sections from the manuscript (include quotes in quotation marks "like this" to indicate direct quotes from your manuscript), or elaborate on this item by providing additional information not in the ms, or briefly explain why the item is not applicable/relevant for your study

"In the subgroup analyses including only participants with poor sleep at baseline, a significant group × time interaction was found for the RIS at post assessment ($F_{1, 180.66}$=12.59; P=<.001; d=.47) and for the perceived insomnia-related impairment at post assessment ($F_{1, 184.45}$=6.69; P=.01; d=.45) as well as for the RIS at follow up assessment ($F_{1, 147.56}$=4.60; P=.03; d=.33). Although the intervention group showed somewhat larger reductions in the PHQ-9 than the control group, the difference was not significant."
"To assess clinically relevant improvements, we assessed how many participants with poor sleep at baseline had reduced their RIS score below 13 points at post-intervention and follow-up. At post-intervention, this was true for N=25 out of 69 (36.2%) assessment completers in the intervention group and N=16 out of 102 (15.7%) assessment completers in the control group. This difference is statistically significant (χ^2=9.531; P=.002). At follow up, there was no significant difference between the groups (21 out of 53 IG completers vs.
25 out of 90 CG completers; χ^2=2.145; P=.143). In the ITT analysis of clinically relevant improvements, the odds of suffering from poor sleep at post-intervention was also significantly larger in the control group than in the intervention group (OR=0.462 [0.218 -0.976])."

19) All important harms or unintended effects in each group
(for specific guidance see CONSORT for harms)

Does your paper address CONSORT subitem 19? *

Copy and paste relevant sections from the manuscript (include quotes in quotation marks "like this" to indicate direct quotes from your manuscript), or elaborate on this item by providing additional information not in the ms, or briefly explain why the item is not applicable/relevant for your study

To our knowledge, there were no important harms or unintended effects.

19-i) Include privacy breaches, technical problems

Include privacy breaches, technical problems. This does not only include physical “harm” to participants, but also incidents such as perceived or real privacy breaches [1], technical problems, and other unexpected/unintended incidents. “Unintended effects” also includes unintended positive effects [2].

	1	2	3	4	5	
subitem not at all important	○	○	○	○	○	essential

Does your paper address subitem 19-i?

Copy and paste relevant sections from the manuscript (include quotes in quotation marks "like this" to indicate direct quotes from your manuscript), or elaborate on this item by providing additional information not in the ms, or briefly explain why the item is not applicable/relevant for your study

There were no privacy breaches or technical problems.

19-ii) Include qualitative feedback from participants or observations from staff/researchers

Include qualitative feedback from participants or observations from staff/researchers, if available, on strengths and shortcomings of the application, especially if they point to unintended/unexpected effects or uses. This includes (if available) reasons for why people did or did not use the application as intended by the developers.

	1	2	3	4	5	
subitem not at all important	○	○	○	○	○	essential

Does your paper address subitem 19-ii?

Copy and paste relevant sections from the manuscript (include quotes in quotation marks "like this" to indicate direct quotes from your manuscript), or elaborate on this item by providing additional information not in the ms, or briefly explain why the item is not applicable/relevant for your study

We did not include qualitative feedback, but asked the participants to complete a short session rating at the end of each intervention chapter.
"Participants who answered the session rating of the intervention chapters rated chapters 1-6 as "good" on average, whereas chapter 7-8 were rated as "moderate". There was no significant difference in session ratings between participants with poor vs. good sleep, except for session 2 that was rated better and nearly "excellent" by participants with good sleep."

DISCUSSION

22) Interpretation consistent with results, balancing benefits and harms, and considering other relevant evidence

NPT: In addition, take into account the choice of the comparator, lack of or partial blinding, and unequal expertise of care providers or centers in each group

22-i) Restate study questions and summarize the answers suggested by the data, starting with primary outcomes and process outcomes (use)

Restate study questions and summarize the answers suggested by the data, starting with primary outcomes and process outcomes (use).

	1	2	3	4	5	
subitem not at all important	○	○	○	○	○	essential

Does your paper address subitem 22-i? *

Copy and paste relevant sections from the manuscript (include quotes in quotation marks "like this" to indicate direct quotes from your manuscript), or elaborate on this item by providing additional information not in the ms, or briefly explain why the item is not applicable/relevant for your study

"The aim of the present study was to evaluate an unguided app-based training to improve sleep quality (insomnia symptoms) in individuals who wish to improve their sleep compared to a waitlist control group. Specifically, we examined participant adherence to the intervention, working alliance and intervention acceptance, and intervention effects on sleep quality (insomnia symptoms), insomnia-related impairment and depression symptoms."

"In our study, the unguided app-based intervention was associated with a short-term improvement of insomnia symptoms that remained stable over a period of six months. Participants in the waitlist condition also reported an improvement in their insomnia symptoms, but this improvement took longer. Effects were more pronounced in individuals with low sleep at baseline. We detected no effect on depression symptoms in our sample, which may largely be due to low average symptom load at baseline."

"Non-usage attrition was comparable to other digital interventions [86], with a larger loss of participants during the early intervention phase and about one in three participants completing the intervention. About one in three participants who were allocated to the intervention group never accessed the intervention."

"Participants in the intervention group were able to form a moderate working alliance with the unguided intervention, with a slightly larger concordance regarding goals than regarding tasks."

"The first six chapters of the intervention were on average rated “good”, while the final two chapters were rated “moderate”."

22-ii) Highlight unanswered new questions, suggest future research

Highlight unanswered new questions, suggest future research.

	1	2	3	4	5	
subitem not at all important	○	○	○	○	○	essential

Does your paper address subitem 22-ii?

Copy and paste relevant sections from the manuscript (include quotes in quotation marks "like this" to indicate direct quotes from your manuscript), or elaborate on this item by providing additional information not in the ms, or briefly explain why the item is not applicable/relevant for your study

"It is noteworthy however that one in four women and one in two men who were assigned to the intervention group did not start the intervention. In clinical practice, the motivation and ability to adhere to a self-guided intervention should thus be carefully assessed.
In participants who did start the intervention, adherence was comparable to other digital interventions. In future adaptations of the interventions, adherence may be improved by involving a multidisciplinary team of psychologists, user experience specialists and UX designers in the design of the user interface, which unfortunately was not possible in our study due to budget constraints."

20) Trial limitations, addressing sources of potential bias, imprecision, and, if relevant, multiplicity of analyses

20-i) Typical limitations in ehealth trials

Typical limitations in ehealth trials: Participants in ehealth trials are rarely blinded. Ehealth trials often look at a multiplicity of outcomes, increasing risk for a Type I error. Discuss biases due to non-use of the intervention/usability issues, biases through informed consent procedures, unexpected events.

	1	2	3	4	5	
subitem not at all important	○	○	○	○	○	essential

Does your paper address subitem 20-i? *

Copy and paste relevant sections from the manuscript (include quotes in quotation marks "like this" to indicate direct quotes from your manuscript), or elaborate on this item by providing additional information not in the ms, or briefly explain why the item is not applicable/relevant for your study

"Limitations of our study include the lack of more specific measures for insomnia-related impairment and socioeconomic consequences including absenteeism or loss of productivity as well as an objective measure of sleep quality. However, subjective measures of sleep quality and insomnia symptoms are widely used in research on CBT-I and the added value of objective measures is a matter of debate [93]. The participants in this trial might have also suffered from sleep disorders other than insomnia , in which an intervention covering the principles of CBT-I is likely to have limited or no effect. Future studies may benefit from including insomnia diagnosis confirmed by a clinician. Adherence to the intervention was approximated by examining usage data. We did not collect information on whether and how participants in the intervention group implemented the suggested behavior changes. Also, the study design is not suitable to determine to what extent the interventions components contributed to the effects."

21) Generalisability (external validity, applicability) of the trial findings

NPT: External validity of the trial findings according to the intervention, comparators, patients, and care providers or centers involved in the trial

21-i) Generalizability to other populations

Generalizability to other populations: In particular, discuss generalizability to a general Internet population, outside of a RCT setting, and general patient population, including applicability of the study results for other organizations

	1	2	3	4	5	
subitem not at all important	○	○	○	○	○	essential

Does your paper address subitem 21-i?

Copy and paste relevant sections from the manuscript (include quotes in quotation marks "like this" to indicate direct quotes from your manuscript), or elaborate on this item by providing additional information not in the ms, or briefly explain why the item is not applicable/relevant for your study

Meine Antwort

21-ii) Discuss if there were elements in the RCT that would be different in a routine application setting

Discuss if there were elements in the RCT that would be different in a routine application setting (e.g., prompts/reminders, more human involvement, training sessions or other co-interventions) and what impact the omission of these elements could have on use, adoption, or outcomes if the intervention is applied outside of a RCT setting.

	1	2	3	4	5	
subitem not at all important	○	○	○	○	○	essential

Does your paper address subitem 21-ii?

Copy and paste relevant sections from the manuscript (include quotes in quotation marks "like this" to indicate direct quotes from your manuscript), or elaborate on this item by providing additional information not in the ms, or briefly explain why the item is not applicable/relevant for your study

"It is noteworthy however that one in four women and one in two men who were assigned to the intervention group did not start the intervention. In clinical practice, the motivation and ability to adhere to a self-guided intervention should thus be carefully assessed."

OTHER INFORMATION

23) Registration number and name of trial registry

Does your paper address CONSORT subitem 23? *

Copy and paste relevant sections from the manuscript (include quotes in quotation marks "like this" to indicate direct quotes from your manuscript), or elaborate on this item by providing additional information not in the ms, or briefly explain why the item is not applicable/relevant for your study

The study was registered prospectively in the ISRCTN database (ref: ISRCTN53553517).

24) Where the full trial protocol can be accessed, if available

Does your paper address CONSORT subitem 24? *

Cite a Multimedia Appendix, other reference, or copy and paste relevant sections from the manuscript (include quotes in quotation marks "like this" to indicate direct quotes from your manuscript), or elaborate on this item by providing additional information not in the ms, or briefly explain why the item is not applicable/relevant for your study

No trial protocol available.

25) Sources of funding and other support (such as supply of drugs), role of funders

Does your paper address CONSORT subitem 25? *

Copy and paste relevant sections from the manuscript (include quotes in quotation marks "like this" to indicate direct quotes from your manuscript), or elaborate on this item by providing additional information not in the ms, or briefly explain why the item is not applicable/relevant for your study

"The Faculty of Psychology at TU Dresden provided funding to cover the study registration and the data management support (MK201805)."

X27) Conflicts of Interest (not a CONSORT item)

X27-i) State the relation of the study team towards the system being evaluated

In addition to the usual declaration of interests (financial or otherwise), also state the relation of the study team towards the system being evaluated, i.e., state if the authors/evaluators are distinct from or identical with the developers/sponsors of the intervention.

	1	2	3	4	5	
subitem not at all important	○	○	○	○	○	essential

Does your paper address subitem X27-i?

Copy and paste relevant sections from the manuscript (include quotes in quotation marks "like this" to indicate direct quotes from your manuscript), or elaborate on this item by providing additional information not in the ms, or briefly explain why the item is not applicable/relevant for your study

Meine Antwort

About the CONSORT EHEALTH checklist

As a result of using this checklist, did you make changes in your manuscript? *

- ○ yes, major changes
- ● yes, minor changes
- ○ no

What were the most important changes you made as a result of using this checklist?

Meine Antwort

How much time did you spend on going through the checklist INCLUDING making changes in your manuscript *

about two to three hours.

As a result of using this checklist, do you think your manuscript has improved? *

- (x) yes
- ( ) no
- ( ) Sonstiges:

Would you like to become involved in the CONSORT EHEALTH group?

This would involve for example becoming involved in participating in a workshop and writing an "Explanation and Elaboration" document

- ( ) yes
- ( ) no
- ( ) Sonstiges:

Any other comments or questions on CONSORT EHEALTH

Meine Antwort

STOP - Save this form as PDF before you click submit

To generate a record that you filled in this form, we recommend to generate a PDF of this page (on a Mac, simply select "print" and then select "print as PDF") before you submit it.

When you submit your (revised) paper to JMIR, please upload the PDF as supplementary file.

Don't worry if some text in the textboxes is cut off, as we still have the complete information in our database. Thank you!

Final step: Click submit !

Click submit so we have your answers in our database!

Senden

Alle Eingaben löschen

Geben Sie niemals Passwörter über Google Formulare weiter.

Dieser Inhalt wurde nicht von Google erstellt und wird von Google auch nicht unterstützt. Missbrauch melden - Nutzungsbedingungen - Datenschutzerklärung

Google Formulare
