# Supplementary material for: Effectiveness of an App-Based Short Intervention to Improve Sleep: Randomized Controlled Trial
Source: JMIR Ment Health. 2023 Mar 21;10:e39052. doi: 10.2196/39052 (PMC10131838; doi:10.2196/39052)
Supplement: Multimedia Appendix 2 [file mental_v10i1e39052_app2.pdf]

Multimedia Appendix 2. Screenshots of the Refresh intervention

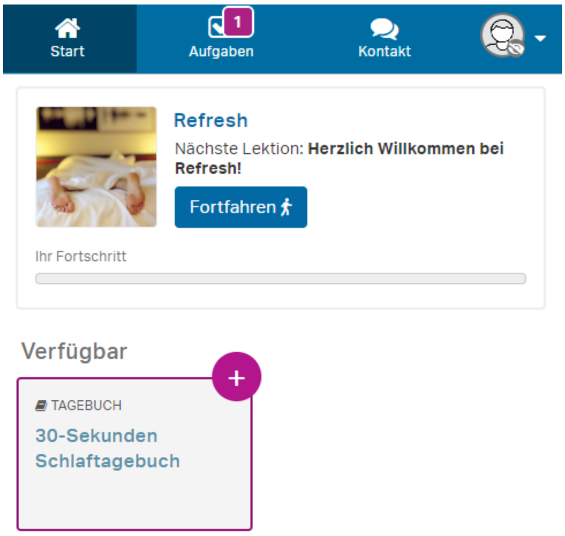

Home screen

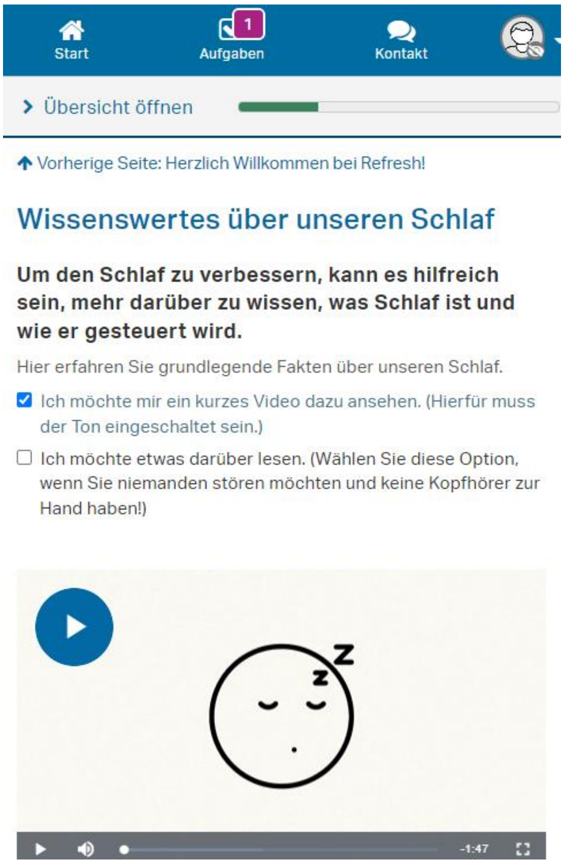

Content page with video or plain text (choice)

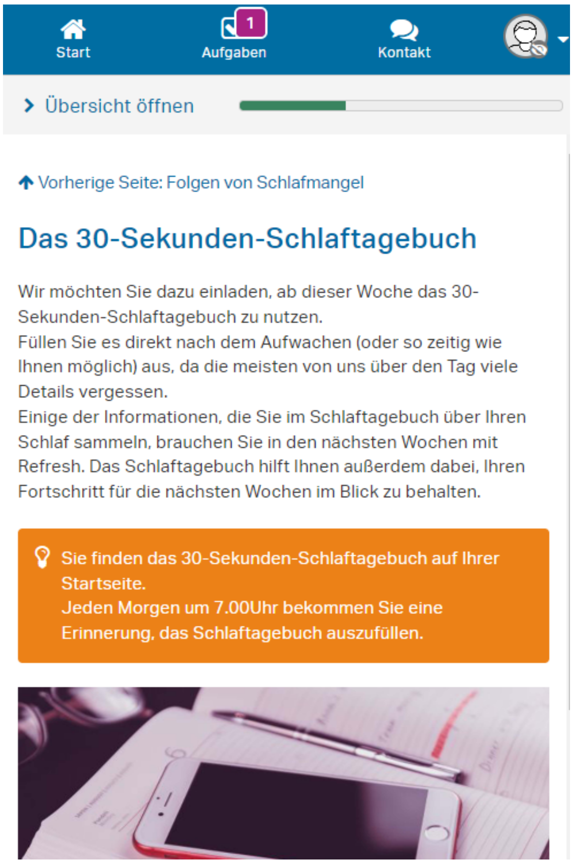

Introduction of the sleep diary

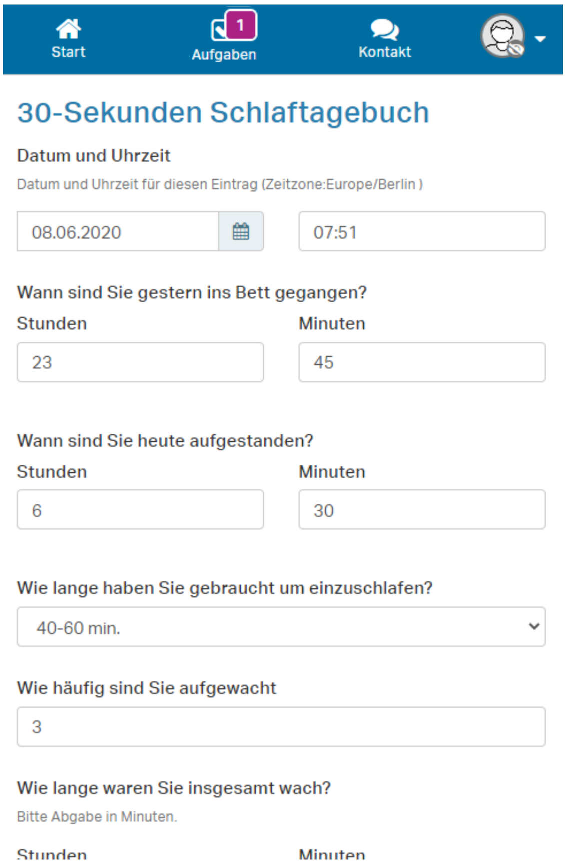

Filling in the sleep diary (example)

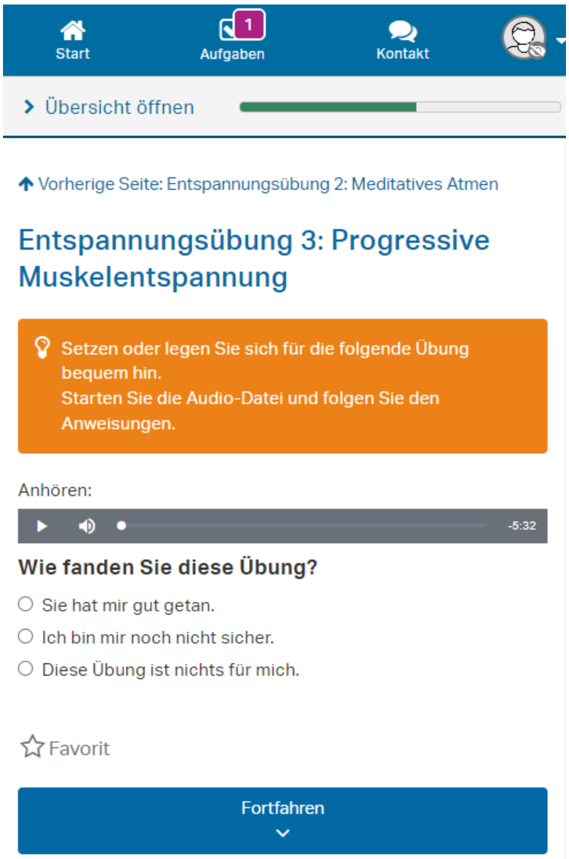

Relaxation exercise
