# Supplementary material for: Effectiveness of an App-Based Short Intervention to Improve Sleep: Randomized Controlled Trial
Source: JMIR Ment Health. 2023 Mar 21;10:e39052. doi: 10.2196/39052 (PMC10131838; doi:10.2196/39052)
Supplement: Multimedia Appendix 3 [file mental_v10i1e39052_app3.pdf]

Multimedia Appendix 3.

Table. Baseline characteristics of participants in the intervention group and the control group.

Characteristics	Full sample (N=371)	Intervention group (n=186)	Control group (n=185)	*P* value
Female gender, n (%)	233 (62.8)	121 (65.1)	112 (60.5)	.368
In a relationship, n (%)	271 (73.0)	128 (68.8)	143 (77.3)	.066
Children <18 years, n (%)	89 (24.0)	42 (22.6)	47 (25.4)	.525
Higher education[a], n (%)	324 (87.3)	162 (87.1)	162 (87.6)	.886
In education, n (%)	122 (32.9)	61 (32.8)	61 (33.0)	.971
Self-employed, n (%)	26 (7.0)	9 (4.8)	17 (9.2)	.101
Shift work, n (%)	34 (9.2)	16 (8.6)	18 (9.7)	.707
In treatment, n (%)	41 (11.1)	18 (9.7)	23 (12.4)	.397
Medication, n (%)	58 (15.6)	26 (14.0)	32 (17.3)	.379
Sick leave, n (%)	14 (3.8)	6 (3.2)	8 (4.3)	.579
Age, mean (SD)	37.30 (14.24)	37.02 (14.43)	37.59 (14.08)	.696
RIS[b] at baseline, mean (SD)	15.33 (6.24)	15.11 (6.34)	15.55 (6.15)	.499
Perceived insomnia-related impairment, mean (SD)	47.23 (27.37)	45.66 (27.52)	48.81 (27.20)	.268
PHQ-9[c] score at baseline, mean (SD)	7.44 (3.56)	7.41 (3.40)	7.46 (3.72)	.891

[a]At least level 4 according to the European Qualifications Framework.

[b]RIS: Regensburg Insomnia Scale.

[c]PHQ-9: 9-item Patient Health Questionnaire (Depression).
